# Supplementary material for: Mutational signatures reveal the dynamic interplay of risk factors and cellular processes during liver tumorigenesis
Source: Nat Commun. 2017 Nov 3;8:1315. doi: 10.1038/s41467-017-01358-x (PMC5670220; doi:10.1038/s41467-017-01358-x)
Supplement: Supplementary file 1 — Supplementary Information [file 41467_2017_1358_MOESM1_ESM.pdf]

a

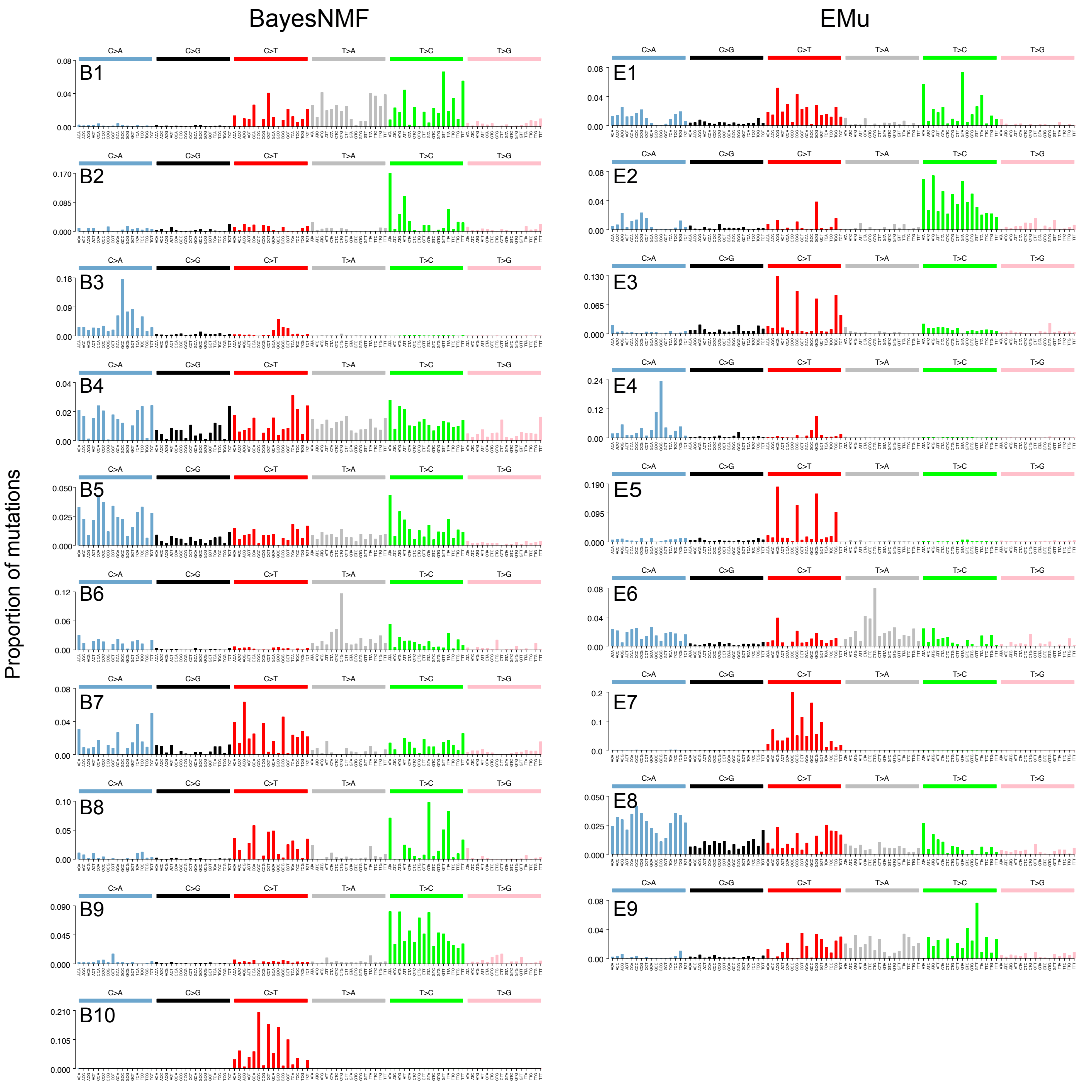

b

Similarities between signatures extracted from this study and previously published (COSMIC)

Signature ID (COSMIC)	BayesNMF equivalent	Cosine similarity (BayesNMF)	EMu equivalent	Cosine similarity (EMu)
Signature 1	B7	0.76	E3,E5	0.93,0.97
Signature 4	B5	0.85	E8	0.81
Signature 5	B4	0.88	-	-
Signature 12	B9	0.92	E2	0.86
Signature 16	B2	0.83	-	-
Signature 22	B6	0.81	E6	0.75
Signature 23	B10	1	E7	0.97
Signature 24	B3	0.92	E4	0.64

Similarities between NOVEL signatures extracted from this study

Signature ID	BayesNMF	EMU	Cosine similarity
Signature.N1	B1	E9	0.92
Signature.N2	B8	E1	0.79

Supplementary Figure 1. *De novo* mutational signature analysis in 44 liver tumors. (**a**) Mutational signatures identified by BayesNMF (n=10) and EMu (n=9) in our new series of 44 liver tumors. (**b**) Correspondance between BayesNMF, EMu and COSMIC signatures. 8/10 BayesNMF and 7/9 EMu signatures correspond to known signatures already described in COSMIC. The two remaining signatures (N1 and N2) are new and similar between the two methods.

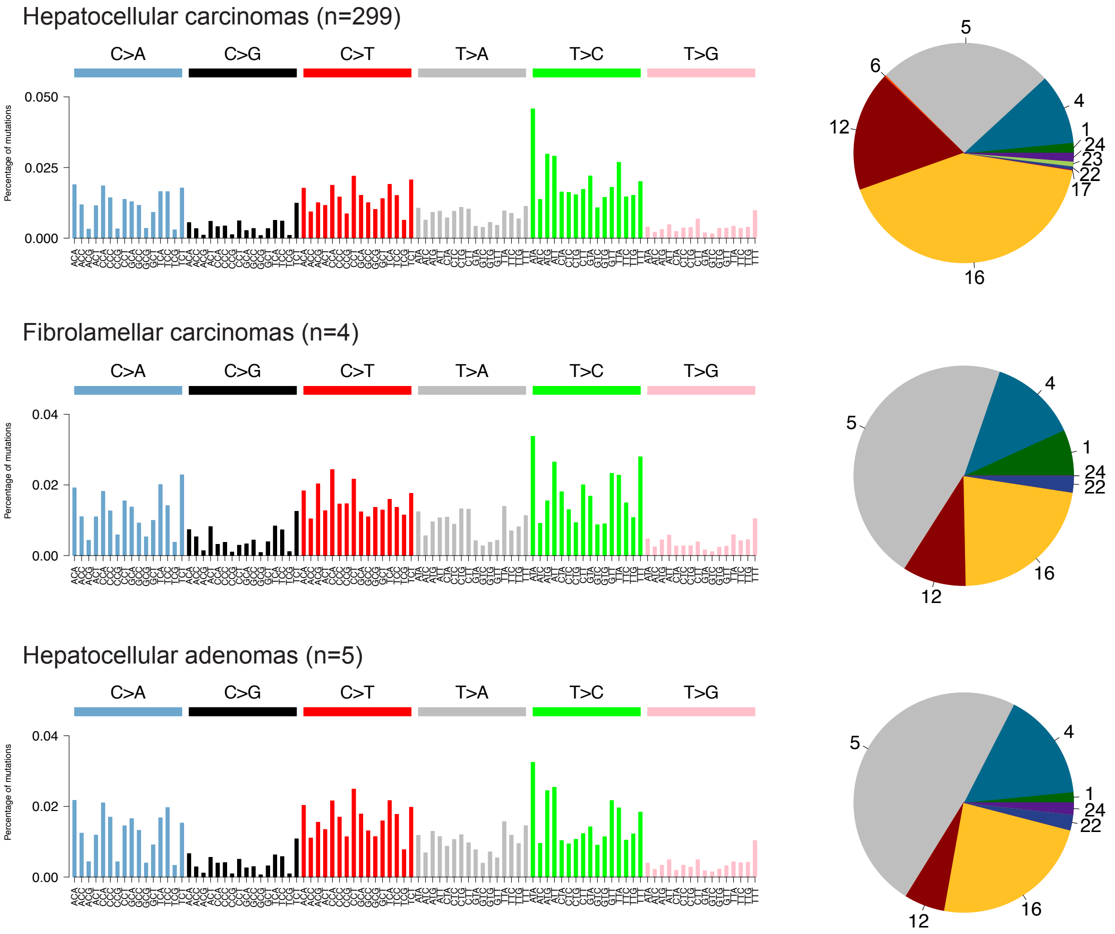

Supplementary Figure 2. Comparison of mutational signatures between histological subtypes. For each tumor type, the average mutational spectrum in 96 categories is represented (left) together with the average decomposition in mutational signatures (right).

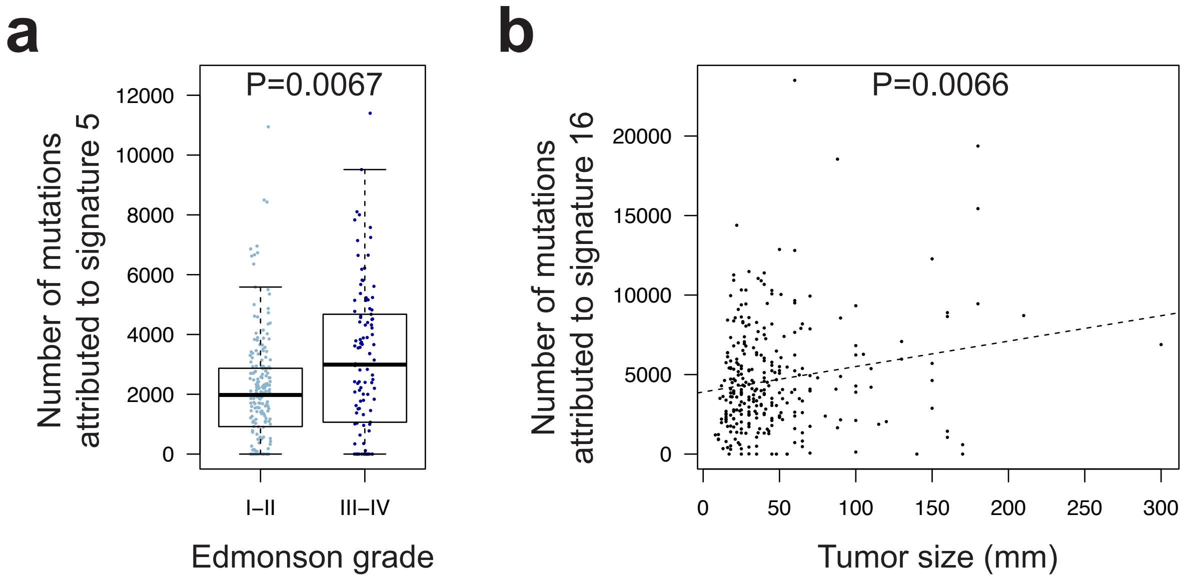

Supplementary Figure 3: Correlation of mutational signatures with clinicopathological features. We assessed correlations between the amount of mutations attributed to each mutational signature and clinicopathological features (fibrosis stage, tumor size, Edmonson grade and vascular invasion). Only two significant associations were detected between signature 5 and Edmonson grade (**a**) and between signature 16 and tumor size (**b**).

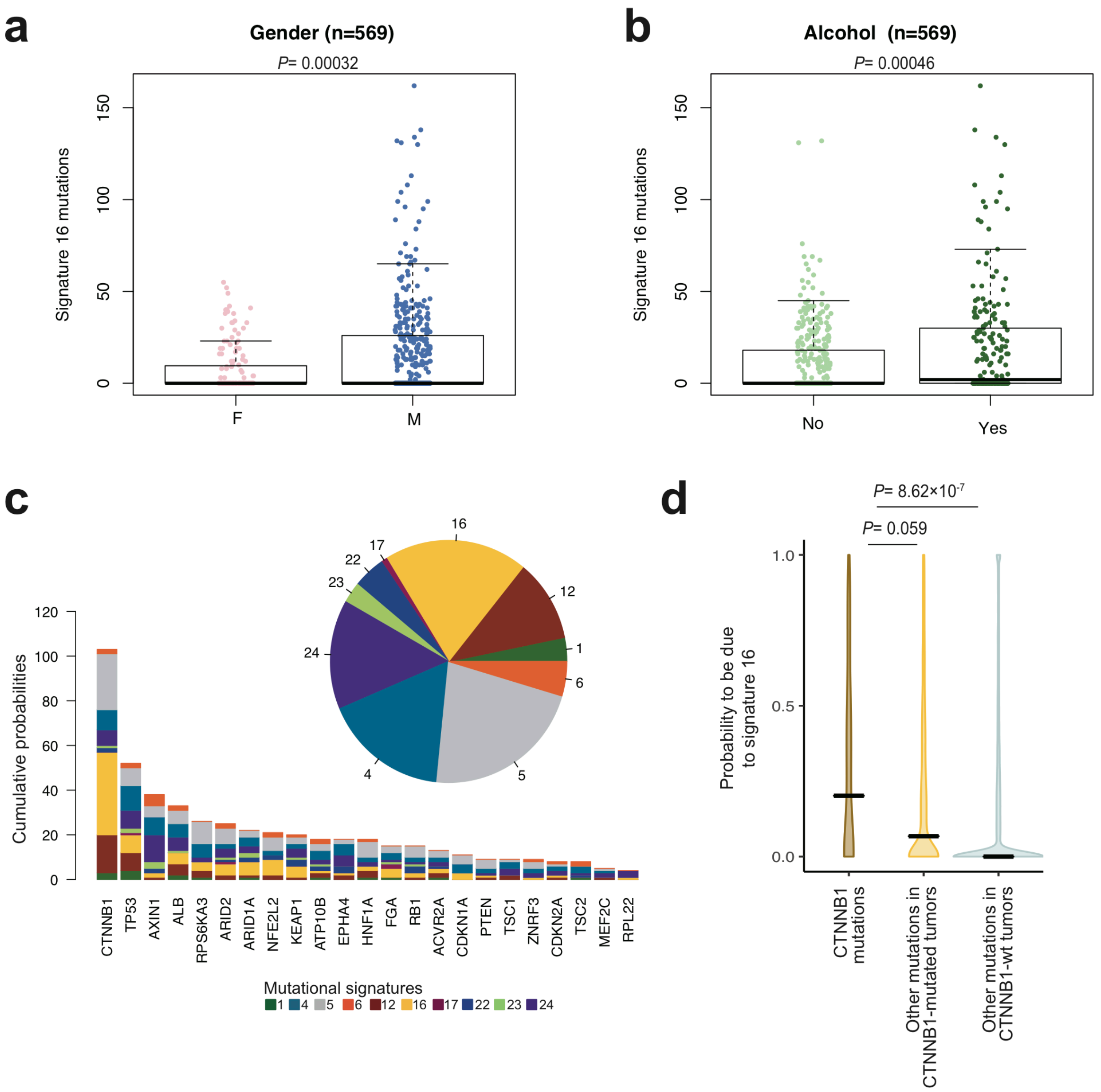

Supplementary Figure 4: Validation of correlations between mutational signatures, risk factors and driver genes in an independent whole exome series. (**a**) Association between the number of mutations attributed to mutational signature 16 and gender. (**b**) Association between the number of mutations attributed to mutational signature 16 and alcohol consumption. (**c**) Distribution of mutational signatures associated with driver gene mutations. We estimated the probability of each driver gene mutation being due to each mutational process. We then summed these probabilities over all mutations and signatures to obtain the cumulative probabilities across all driver gene mutations (pie chart) and for each driver gene separately (barplot). (**d**) *CTNNB1* mutations (left) overall have higher probabilities being due to signature 16 than other mutations in the same samples (middle) and in other samples (right). The violin plots represent the distribution of probabilities for each group of mutations and horizontal segments highlight median values.

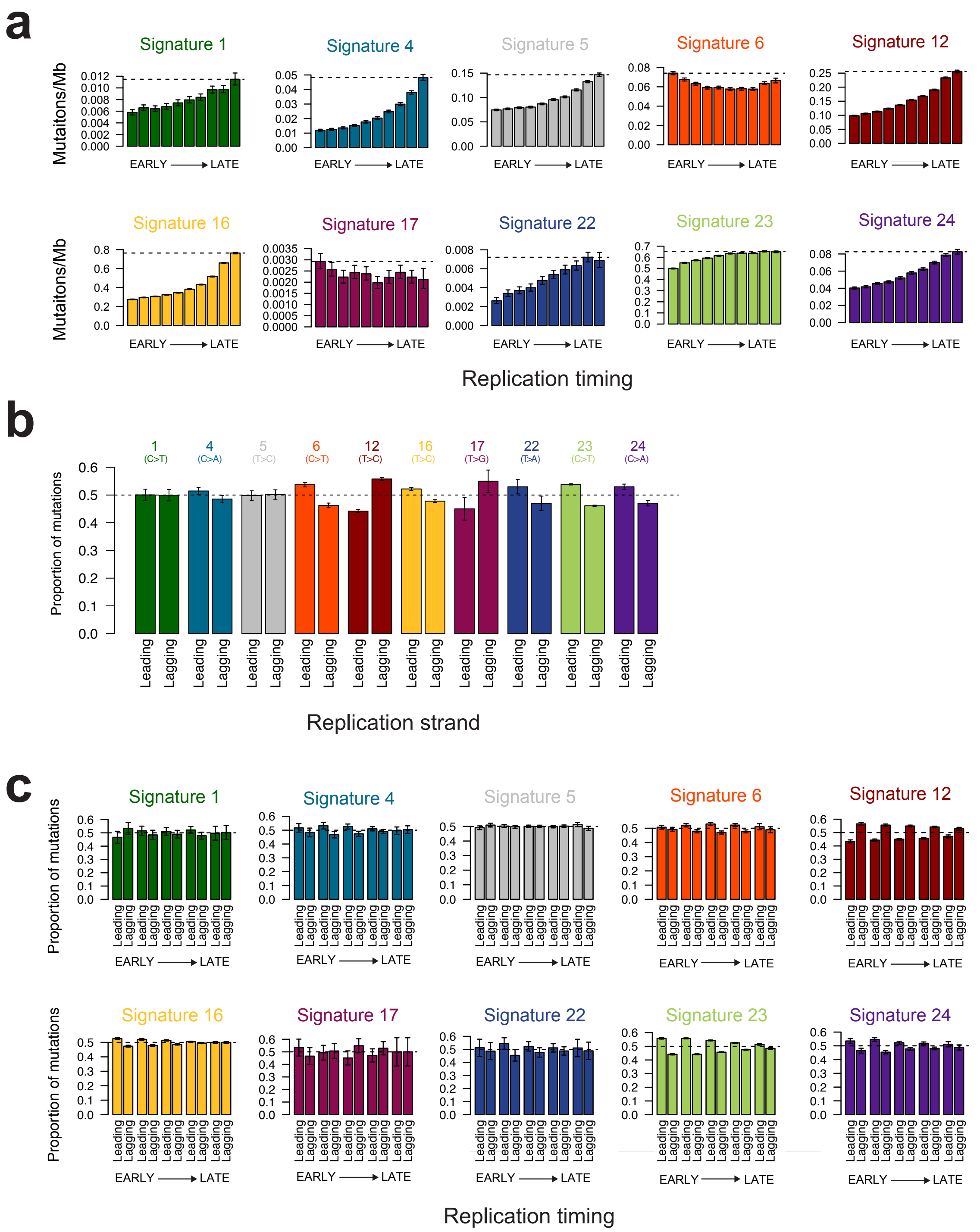

Supplementary Figure 5. DNA replication modulates the activity of mutational signatures in HCC. (**a**) Correlation between replication timing and SNV rates broken down by mutational signature. Genomic regions were classified in ten replication deciles using RepliSeq data generated by the ENCODE project for the HepG2 cell line. The mutation rate was calculated within each decile, considering only mutations attributed to a given signature with probability ≥0.7. (**b**) Analysis of replicative strand asymmetries. The proportions of mutations occurring on the leading and lagging strand are represented for each mutational signature. Only the dominant substitution type (indicated in parentheses) was considered for each signature. (**c**) Replicative strand asymmetry was analyzed separately within each replication timing decile. The asymmetry between the two strands is stronger in early replicating regions.

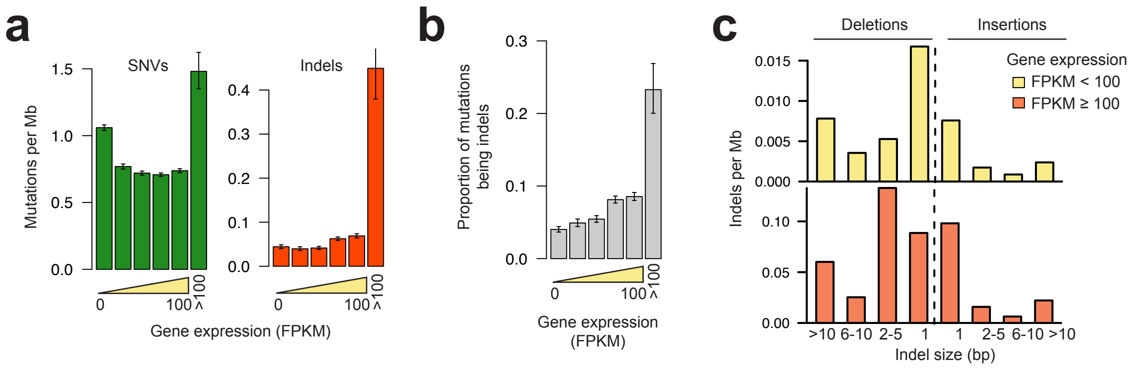

Supplementary Figure 6: Validation of the high amount of indels associated with very highly expressed genes in an independent whole exome series. (**a**) Number of single nucleotide variants (SNVs, left) and small insertions and deletions (indels, right) per megabase in genes as a function of expression level. Genes with expression between 0 and 100 fragments per kilobase of exons per million reads (FPKM) were divided in 5 gene expression quintiles. A separate group was created for very highly expressed genes (FPKM≥100). Error bars indicate the 95% confidence intervals of the estimated mutation rates. (**b**) Proportion of mutations being indels as a function of expression level. For each gene expression group, the proportion of mutations being indels was estimated as the number of indels divided by the number of SNVs + indels in genes belonging to the expression group. Error bars indicate the 95% confidence intervals of the estimated mutation rates. (**c**) Distribution of indel types and sizes in very highly expressed (FPKM≥100) versus all other genes.

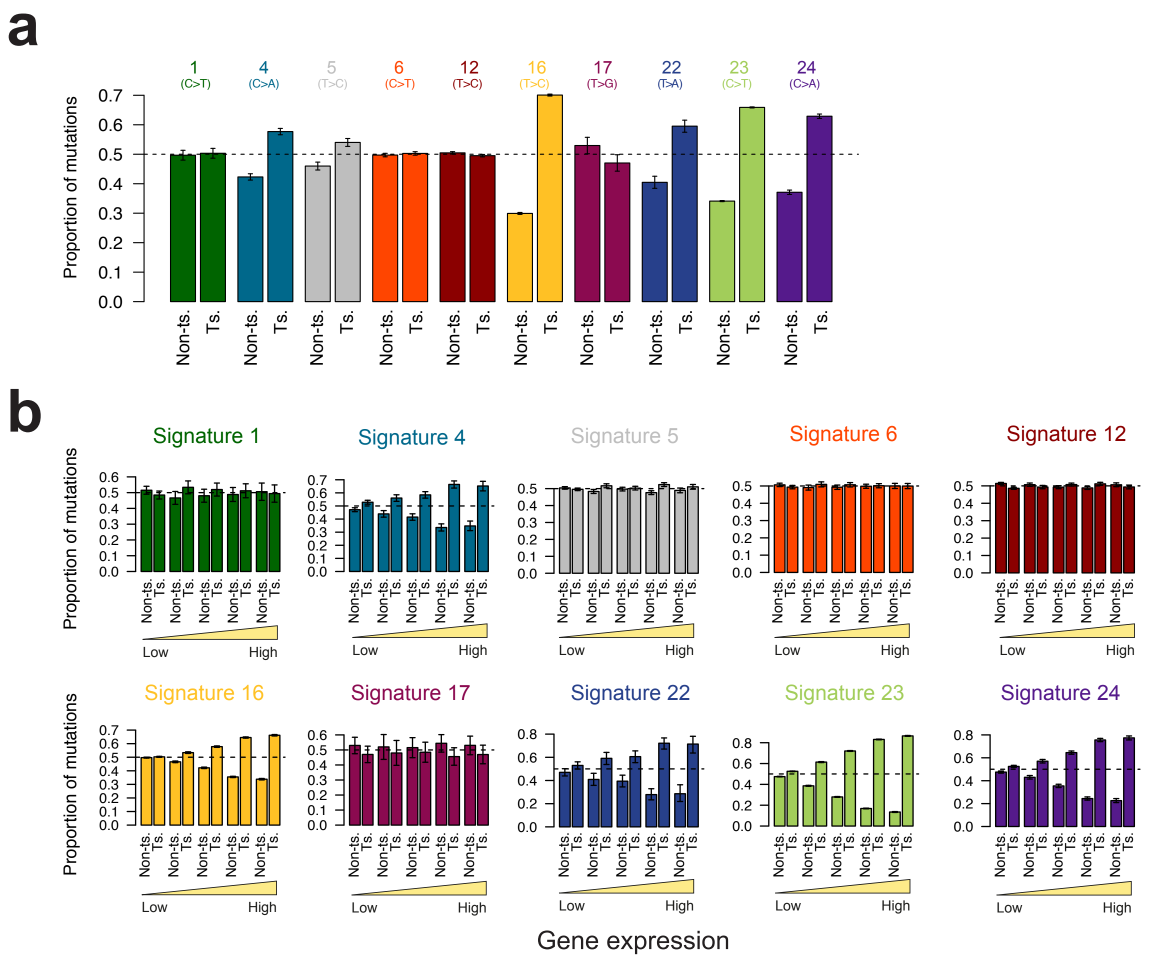

Supplementary Figure 7. Transcriptional strand biases associated with liver cancer mutational signatures (**a**) Analysis of transcriptional strand asymmetries. The proportions of mutations occurring on the transcribed (Ts.) and non-transcribed (Non-ts.) strand are represented for each mutational signature. Only the dominant substitution type (indicated in parentheses) was considered for each signature. (**b**) Transcriptional strand asymmetry as a function of gene expression. Genes were split in 5 quintiles from low to high expression and the transcriptional strand asymmetry was estimated within each group. The asymmetry between the two strands is stronger in highly expressed genes.

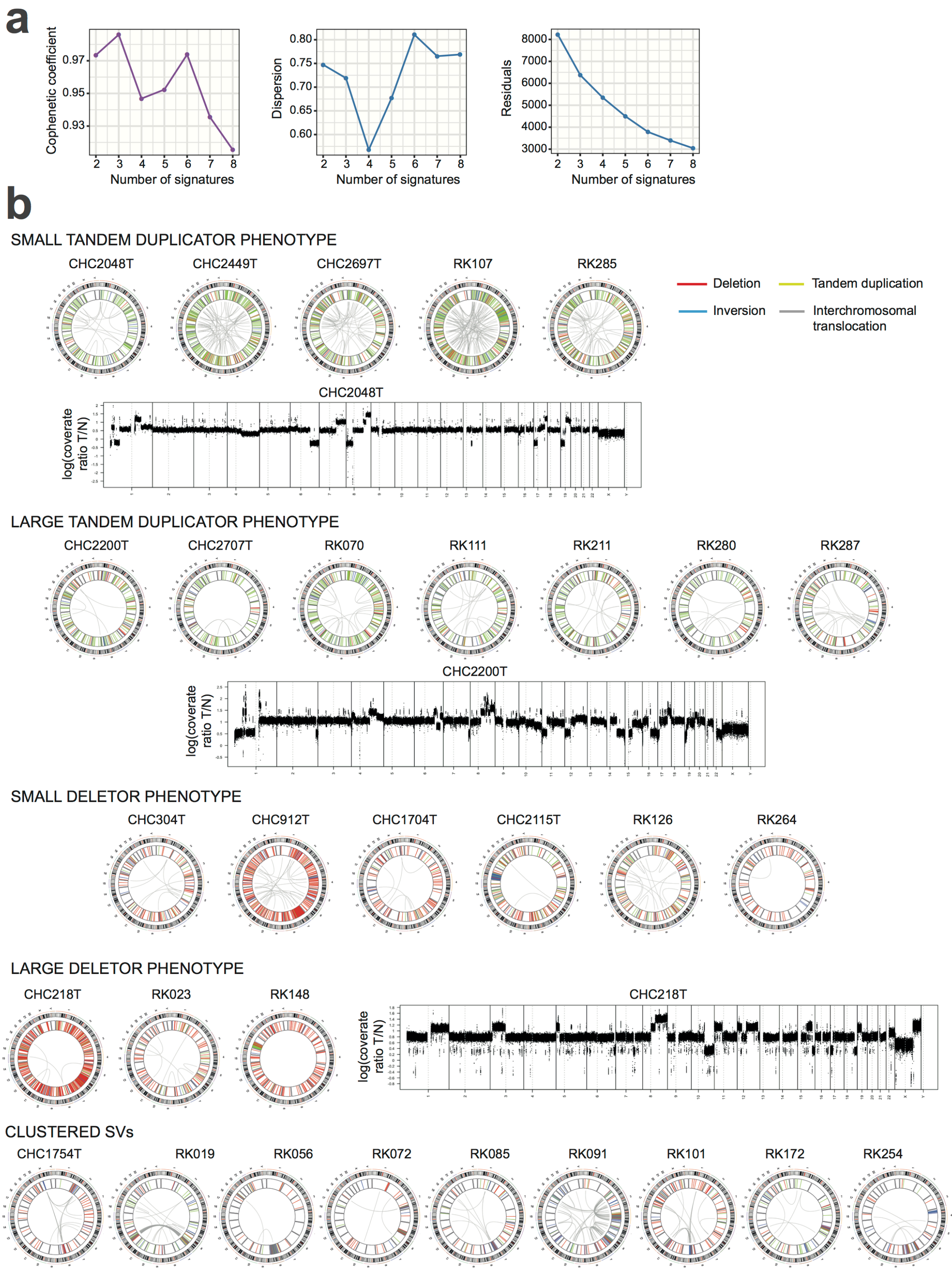

Supplementary Figure 8. Structural rearrangement signature analysis. (**a**) Non-negative matrix factorization (NMF) metrics used to determine the optimal number of signatures. With 6 signatures, we obtain good cophenetic coefficient and the best dispersion score. (**b**) Structural rearrangement profiles of tumors displaying specific phenotypes are represented with CIRCOS plots. Coverage log-ratio profiles from 3 representative tumors also show the accumulation of deletions and duplications of a specific size.

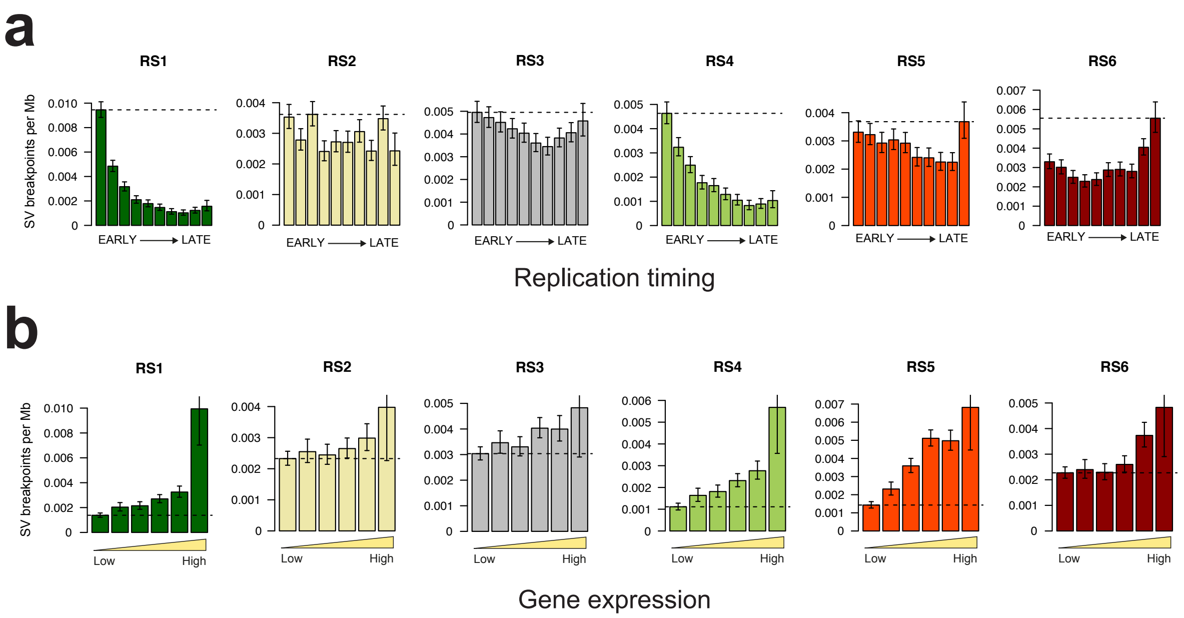

Supplementary Figure 9. DNA replication and gene expression modulate the activity of structural rearrangement signatures. (**a**) Correlation between replication timing and SV breakpoint rates broken down by SV signature. (**b**) Correlation between gene expression and SV breakpoint rates broken down by SV signature.

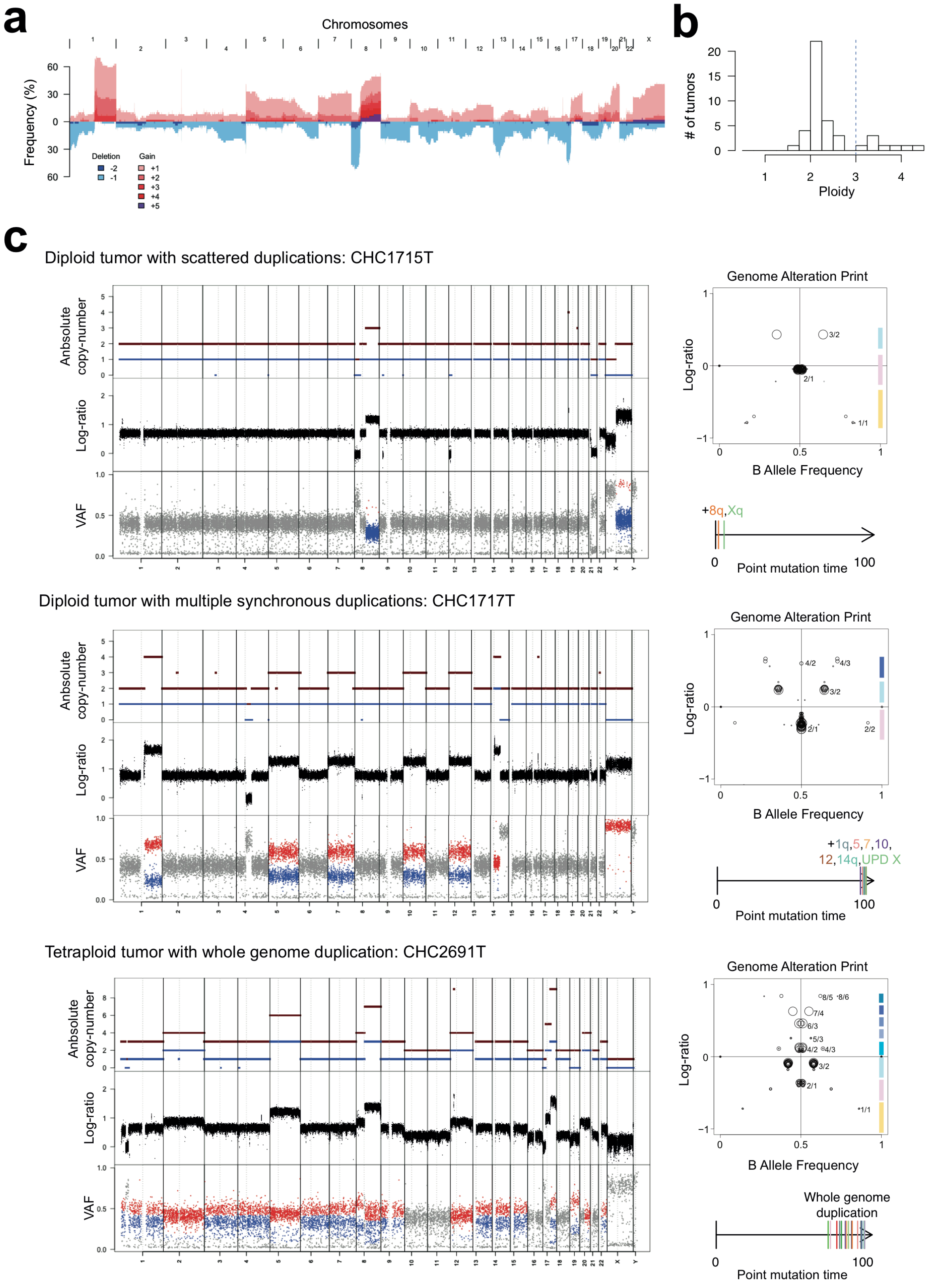

Supplementary Figure 10: Copy-number analysis and duplication timing. (**a**) Frequency of gains and deletions along the genome. The number of copies above (gains) or below (deletions) ploidy is indicated with a color code. (**b**) Distribution of ploidy across the cohort. (**c**) Representative examples of a diploid tumor with scattered

duplications (top), a diploid tumor with multiple synchronous duplications (middle) and a tetraploid tumor with whole genome duplication (bottom). Absolute copy numbers (total: red, minor allele: blue) and coverage log-ratio between tumor and normal are represented along the genome. The VAF (Variant Allele Fraction) plot represents the proportion of mutated reads for each somatic mutation. Duplicated mutations are indicated in red and non-duplicated mutations in blue. The ratio of duplicated over non-duplicated mutations was used to time duplications as explained in Online Methods. Tumor CHC1715T displays early duplications with few duplicated mutations. By contrast, tumors CHC1717T and CHC2691T have similar numbers of duplicated and non-duplicated mutations, indicating that these events occurred very late in tumor history. Note the similar ratios of duplicated and non-duplicated mutations indicating synchronous acquisition of multiple gains. The Genome Alteration Print (GAP) patterns on the right side are sideview projections of segmented log ratio (y axis) and B Allele Frequency (x axis) used to determine visualize absolute copy-numbers and tumor ploidy (Popova et al., Genome Biol 2009). Each circle represents a chromosome segment and is designated by the ratio of copy-number to most abundant allele counts (e.g. 3/2 indicates that the segment has a total copy number of 3, with 2 copies of one allele, and 1 of the other).

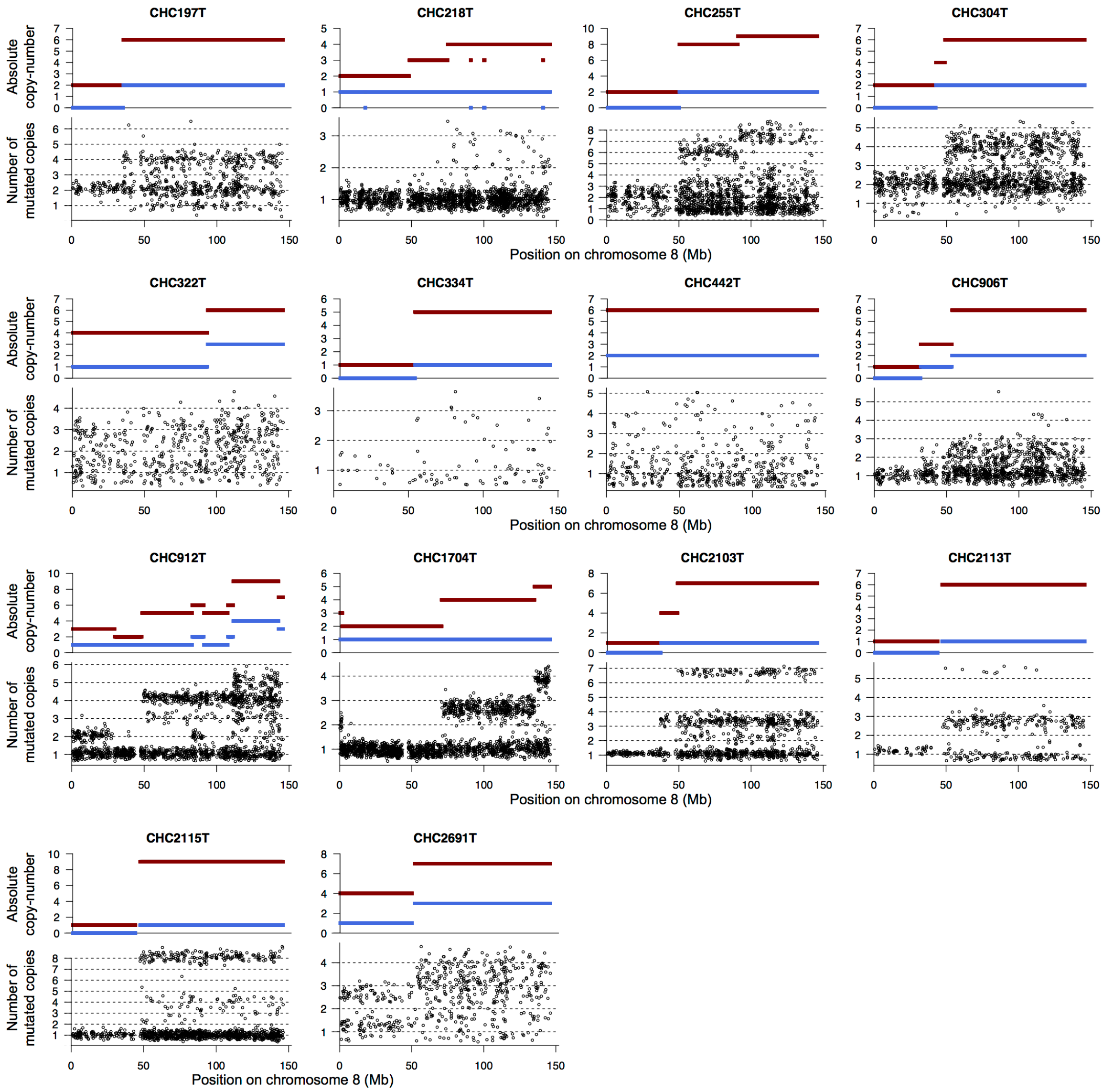

Supplementary Figure 11: Chromosome 8q gains are acquired and selected repeatedly along tumorigenesis. Fourteen out of 44 tumors displayed ≥2 extra-copies of chromosome arm 8q in our series. For each tumor, the top panel represents the total copy-number (red) and the copy-number of the minor allele (blue) along chromosome 8. The bottom panel indicates the estimated number of chromosomes harboring each somatic mutation (multiplicity), estimated as explained in Online Methods. Most cases display several levels of multiplicities, indicating that the total copy-number was not achieved in a single step but through several rounds of duplication.

Supplementary Table 1: Clinical annotations

Tumor ID	Tumor type	Gender	Age	Geographic origin	Alcohol Intake	Hepatitis B	Hepatitis C	Hemochromatosis	Metabolic syndrome	Tobacco	Without etiology	Edmonson grade	Fibrosis stage (METAVIR)	Largest nodule Diameter (mm)	Satellite nodules	Vascular Invasion
CHC018T	HCC	F	35	Africa	no	yes	no	no	no	no	no	III	F2	170	no	yes
CHC1704T	HCC	M	44	Africa	no	yes	no	no	no	no	no	III	F3	140	yes	yes
CHC1715T	HCC	M	72	Europe	yes	no	no	no	no	yes	no	II	F1	60	no	no
CHC1717T	HCC	M	51	Africa	no	yes	no	no	no	N.d.	no	II	F4	55	yes	yes
CHC1754T	HCC	M	35	Africa	no	yes	no	no	no	no	no	IV	F2	170	yes	yes
CHC197T	HCC	M	73	Europe	yes	no	no	no	no	N.d.	no	III	F2	130	yes	yes
CHC2048T	HCC	M	65	Europe	yes	no	no	no	no	no	no	III	F0	100	yes	yes
CHC205T	HCC	M	46	Europe	yes	no	no	no	no	yes	no	III	F0	100	no	no
CHC2103T	HCC	M	56	Europe	yes	no	yes	no	no	yes	no	III	F1	28	yes	yes
CHC2111T	HCC	F	55	Europe	no	no	no	no	no	yes	yes	II	F1	60	no	no
CHC2113T	HCC	M	61	Europe	yes	no	no	no	no	no	no	III	F1	90	no	no
CHC2115T	HCC	M	74	Europe	yes	no	no	no	yes	yes	no	III	F1	100	yes	yes
CHC2132T	HCC	M	56	Europe	no	no	no	no	yes	yes	no	III	F1	180	no	no
CHC218T	HCC	M	69	Europe	no	no	no	no	yes	no	no	III	F1	130	yes	yes
CHC2200T	HCC	M	68	Europe	no	no	no	no	yes	yes	no	IV	F1	110	yes	yes
CHC2443T	HCC	M	73	Europe	yes	no	no	no	yes	yes	no	III	F1	48	no	yes
CHC2448T	HCC	M	81	Europe	no	no	no	no	yes	no	no	II	F1	75	no	yes
CHC2449T	HCC	M	80	Europe	no	no	no	no	yes	yes	no	IV	F0	90	yes	no
CHC2538T	HCC	F	75	Europe	no	no	no	no	yes	no	no	II	F2	40	no	no
CHC2539T	HCC	F	41	Europe	no	no	no	no	no	yes	yes	III	F0	160	yes	yes
CHC2691T	HCC	M	69	Europe	yes	no	no	no	yes	N.d.	no	III	F1	60	no	no
CHC2697T	HCC	M	64	Europe	yes	no	no	no	no	no	no	IV	F1	110	yes	yes
CHC2707T	HCC	M	78	Europe	no	no	no	yes	no	yes	no	II	F1	30	no	yes
CHC304T	HCC	M	77	Europe	yes	no	no	no	no	yes	no	III	F1	180	no	yes
CHC309T	HCC	F	69	Europe	no	no	yes	no	no	N.d.	no	III	F2	20	no	yes
CHC314T	HCC	M	71	Europe	yes	no	yes	no	no	N.d.	no	II	F2	45	no	no
CHC320T	HCC	M	65	Europe	yes	no	yes	no	no	N.d.	no	III	F4	35	no	no
CHC322T	HCC	M	74	Europe	yes	no	no	no	no	N.d.	no	III	F4	40	no	no
CHC429T	HCC	F	65	Europe	no	no	no	no	no	no	yes	III	F0	45	yes	yes
CHC433T	HCC	M	70	Europe	yes	no	no	no	yes	N.d.	no	II	F1	180	yes	yes
CHC892T	HCC	F	72	Europe	no	no	no	no	no	no	yes	I	F0	55	no	no
CHC909T	HCC	M	70	Europe	no	no	no	no	no	yes	yes	III	F1	210	yes	yes
CHC912T	HCC	M	78	Europe	yes	no	yes	no	no	yes	no	IV	F1	60	yes	yes
CHC255T	FLC	F	39	Europe	no	no	no	no	no	yes	yes	NA	F0	140	yes	no
CHC906T	FLC	F	50	Europe	no	no	no	no	no	N.d.	yes	NA	F1	80	no	no
CHC334T	FLC	F	24	Europe	no	no	no	no	no	N.d.	yes	NA	F1	90	no	no
CHC442T	FLC	F	27	Europe	no	no	no	no	no	yes	yes	NA	F0	100	yes	yes
CHC361TA	HCC in HCA (progresssion of CHC361TB)	F	67	Europe	no	no	no	no	yes	N.d.	no	II	F1	60	no	no
CHC361TB	HCA	F	67	Europe	no	no	no	no	yes	N.d.	no	NA	F1	60	NA	NA
CHC465T	HCC in HCA (progresssion of CHC464T)	F	42	Europe	no	no	no	no	no	yes	yes	I	F0	100	no	no
CHC464T	HCA	F	42	Europe	no	no	no	no	no	yes	yes	NA	F0	120	NA	NA
CHC2432T	HCA	F	43	Europe	no	na	N.d.	no	no	no	yes	NA	F0	15	NA	NA
CHC2446T	HCA	F	45	Europe	no	no	no	no	no	yes	yes	NA	F1	50	NA	NA
CHC605T	HCA	F	47	Europe	no	no	no	no	yes	no	no	NA	F1	60	NA	NA

HCC: Hepatocellular Carcinoma; FLC: Fibrolamellar Carcinoma; HCA: Hepatocellular Adenoma
N.d.: Not determined
NA: Not Applicable (e.g. "Edmonson Grade" is not applicable to HCA)

Supplementary Table 2: Coverage and variant calling statistics

Tumor ID	Sequencing Series	Tumor Coverage	Normal Coverage	# of somatic SNVs	# of somatic indels	# of somatic SVs
CHC1704T	Integragen	76.54	38.97	33509	878	114
CHC1715T	Integragen	92.15	55.57	23661	1076	21
CHC1717T	Integragen	78.22	39.83	17469	795	12
CHC1754T	Integragen	71.07	35.11	20852	844	174
CHC2048T	Integragen	89.95	61.45	13534	650	186
CHC2103T	Integragen	97.52	63.65	16913	836	46
CHC2111T	Integragen	87.53	52.26	6059	278	9
CHC2113T	Integragen	100.52	59.34	7988	450	7
CHC2115T	Integragen	96.37	49.79	15818	818	134
CHC2132T	Integragen	100.66	54.89	27878	2125	46
CHC218T	Integragen	99.47	54.41	21208	1053	497
CHC2200T	Integragen	100.38	47.6	17995	960	191
CHC2432T	Integragen	52.8	58.88	2921	193	1
CHC2443T	Integragen	98.74	42.46	12924	835	102
CHC2446T	Integragen	93.74	49.86	5289	166	4
CHC2448T	Integragen	87.64	55.68	12885	808	20
CHC2449T	Integragen	96.9	55.06	11782	681	494
CHC2538T	Integragen	94.85	56.74	13939	759	22
CHC2539T	Integragen	101.19	57.83	6125	344	33
CHC2691T	Integragen	96.09	57.26	9760	686	14
CHC2697T	Integragen	94.3	58.73	14097	757	314
CHC2707T	Integragen	86.18	46.44	13032	538	93
CHC361TA	Integragen	84.74	56.07	9923	184	9
CHC361TB	Integragen	83.51	55.78	7654	187	10
CHC464T	Integragen	86.31	58.4	19037	325	4
CHC465T	Integragen	84	58.11	14760	626	17
CHC605T	Integragen	90.15	57.88	5748	169	2
CHC892T	Integragen	64.95	37.67	609296	1324	13
CHC909T	Integragen	94.83	49.22	15156	1109	50
CHC912T	Integragen	96.3	53.77	25775	2251	370
CHC018T	CNG	102.02	101.41	7804	747	101
CHC197T	CNG	106.9	90.79	12440	929	16
CHC205T	CNG	108.73	91.99	11307	980	50
CHC255T	CNG	89.31	56.09	23069	754	78
CHC304T	CNG	79.52	93.75	22173	2253	136
CHC309T	CNG	100.63	111.66	9559	558	39
CHC314T	CNG	90.05	116.4	15677	853	143
CHC320T	CNG	94.06	98.8	9907	680	65
CHC322T	CNG	93.62	101.1	10303	511	72
CHC334T	CNG	108.4	113.67	2162	140	13
CHC429T	CNG	108.34	114.29	26459	915	31
CHC433T	CNG	97.84	112.55	35518	1256	70
CHC442T	CNG	98.37	94.44	6603	137	13
CHC906T	CNG	109.02	73.87	19156	624	70

Supplementary Table 3: HBV insertion sites identified in 44 liver tumors

Tumor ID	Virus	Chromosome	Position	Gene
CHC1704T	HBV	chr4	151597758	LRBA
CHC1704T	HBV	chr5	1297642	TERT promoter
CHC1704T	HBV	chr11	45329533	Intergenic
CHC1704T	HBV	chrX	72337840	Intergenic
CHC1717T	HBV	chr5	1295568	TERT promoter
CHC1754T	HBV	chr6	111627366	REV3L

Supplementary Table 4: Comparison of clinical annotations between the INSERM and ICGC-Japan HCC series

Clinical feature		INSERM series (n= 35 HCC)	ICGC-Japan (n=264 HCC)	P-value	Test
Gender	Male	26 (74%)	198 (75%)	1	Fisher's exact test
	Female	9 (26%)	66 (25%)		
Age (median)		69	68	0.59	Wilcoxon rank-sum test
Alcohol	Yes	16 (46%)	105 (42%)	0.72	Fisher's exact test
	No	19 (54%)	145 (58%)		
HBV	Yes	4 (11%)	78 (30%)	0.026 (Fisher's exact test)	Fisher's exact test
	No	31 (89%)	186 (70%)		
HCV	Yes	5 (14%)	149 (56%)	2.59E-06	Fisher's exact test
	No	30 (86%)	115 (44%)		
Tobacco	Yes	15 (58%)	143 (57%)	1	Fisher's exact test
	No	11 (42%)	107 (43%)		
	Missing	9	14		
Fibrosis stage*	F0	0 (0%)	12 (5%)	1.15E-05	Chi-square test
	F1 or F0-F1	25 (71%)	31 (12%)		
	F2 or F1-F2	0 (0%)	66 (25%)		
	F3 or F2-F3	7 (20%)	62 (23%)		
	F4	3 (9%)	93 (35%)		
Tumor size (mm, median)		90	30	3.25E-11	Wilcoxon rank-sum test
Edmonson grade	I-II	11 (31%)	185 (70%)	1.39E-05	Fisher's exact test
	III-IV	24 (69%)	78 (30%)		
	Missing	0	1		
Vascular invasion	Yes	21 (60%)	89 (34%)	0.0047	Fisher's exact test
	No	14 (40%)	171 (66%)		
	Missing	4	0		

* According to METAVIR score for the INSERM series, and the New Inuyama Classification for the ICGC-Japan series.

Supplementary Table 5: Association between mutational signatures and risk factors

Mutational signature	Clinical feature	P-value (univariate)	P-value (multivariate)
Signature 1	Female gender	0.004062173	0.019678568
Signature 4	Age	1.67E-05	6.27E-06
	Tobacco	0.019146798	0.004285109
Signature 6	Female gender	0.01598921	0.01598921
Signature 16	Age	4.35E-05	0.000344604
	Alcohol	2.01E-06	0.004154386
	Male gender	1.53E-06	0.019915826
	Tobacco	4.80E-05	0.04799814

Supplementary Table 6: Subclonal mutations identified in driver genes

Tumor ID	Hugo Symbol	Variant Classification	Genome Change	Protein Change	Depth in non-tumor	Variant allele fraction in non-tumor	Depth in tumor	Variant Allele Fraction in tumor	Cancer Cell Fraction (CCF)	CCF confidence interval - lower bound	CCF confidence interval - upper bound
CHC361TA	PTEN	Missense_Mutation	g.chr10:89720838A>T	p.K330I	46	0	78	0.038461538	0.131	0.027	0.37
CHC361TA	ARID2	Nonsense_Mutation	g.chr12:46205216C>A	p.Y100*	61	0	80	0.0375	0.128	0.027	0.36
CHC1715T	PTEN	Missense_Mutation	g.chr10:89692905G>A	p.R130Q	58	0	114	0.035087719	0.091	0.025	0.227
CHC2443T	CTNNB1	Missense_Mutation	g.chr3:41266097G>A	p.D32N	41	0	99	0.141414141	0.39	0.219	0.623
CHC2443T	CTNNB1	Missense_Mutation	g.chr3:41266101C>G	p.S33C	40	0	102	0.107843137	0.297	0.152	0.509
CHC2443T	CTNNB1	Missense_Mutation	g.chr3:41266097G>T	p.D32T	41	0	99	0.02020202	0.056	0.027	0.788
CHC2132T	FGA	Silent	g.chr4:155507438A>G	p.G381G	59	0	73	0.04109589	0.053	0.011	0.149
CHC2707T	ATP10B	Missense_Mutation	g.chr5:160049420G>T	p.T598N	53	0	66	0.045454545	0.169	0.035	0.472
CHC309T	ALB	Frame_Shift_Del	g.chr4:74284017delCAAG	p.I397fs	128	0	91	0.252747253	0.637	0.422	0.894
CHC433T	TSC2	Silent	g.chr16:2107132C>T	p.H267H	41	0	24	0.083333333	0.172	0.021	0.559
CHC018T	AXIN1	Nonsense_Mutation	g.chr16:396458G>A	p.Q190*	107	0	61	0.836065574	0.827	0.712	0.909
CHC205T	RB1	Missense_Mutation	g.chr13:49039143C>A	p.R741S	38	0	45	0.066666667	0.174	0.036	0.477
