# Supplementary material for: Mutational signatures reveal the dynamic interplay of risk factors and cellular processes during liver tumorigenesis
Source: Nat Commun. 2017 Nov 3;8:1315. doi: 10.1038/s41467-017-01358-x (PMC5670220; doi:10.1038/s41467-017-01358-x)
Supplement: Supplementary file 3 — Description of Additional Supplementary Files [file 41467_2017_1358_MOESM3_ESM.pdf]

## Description of Additional Supplementary Files

File Name: Supplementary Data 1
Description: Structural Rearrangements identified in 44 liver tumors.

File Name: Supplementary Data 2
Description: Natural history of 44 liver tumors. For each tumor, the numbers of clonal and subclonal mutations are indicated as well as the contribution of mutational signatures to each set of mutations. Driver alterations and copy-number alterations in the clonal and subclonal compartments are indicated with a color code indicating the type of event and the most likely signature of origin, as represented in the legend (last page). Copy-number alterations are indicated and duplications are positioned according to their timing in point mutation time. Tumors were classified by etiology and the main clinical characteristics of each patient are indicated.
