# Supplementary material for: Mutational signatures reveal the dynamic interplay of risk factors and cellular processes during liver tumorigenesis
Source: Nat Commun. 2017 Nov 3;8:1315. doi: 10.1038/s41467-017-01358-x (PMC5670220; doi:10.1038/s41467-017-01358-x)
Supplement: Supplementary file 5 — Supplementary Data 2 [file 41467_2017_1358_MOESM5_ESM.pdf]

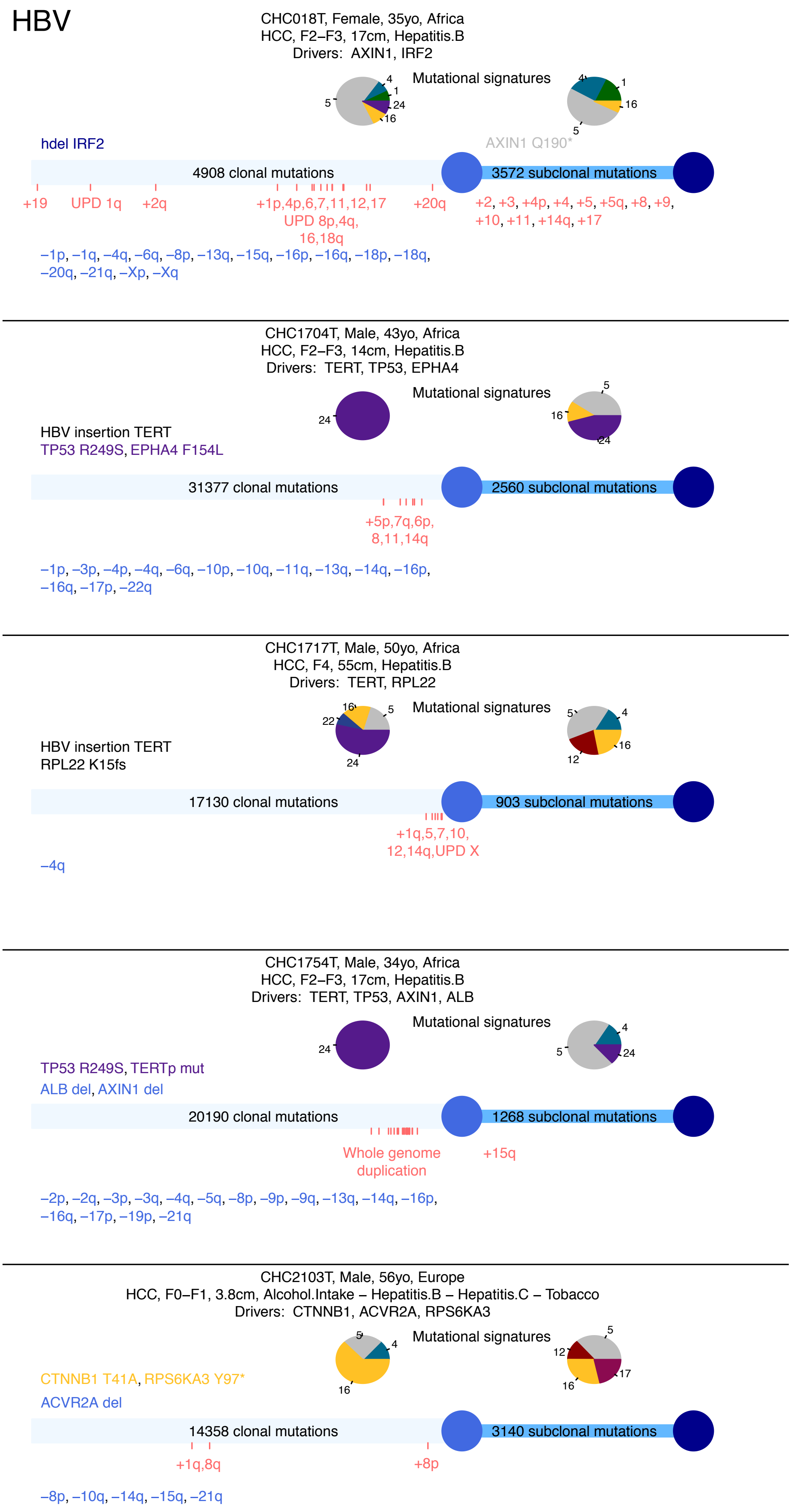

HBV
CHC018T, Female, 35yo, Africa
HCC, F2–F3, 17cm, Hepatitis.B
Drivers: AXIN1, IRF2
Mutational signatures
5
4
1
24
16
hdel IRF2
AXIN1 Q190*
4908 clonal mutations
3572 subclonal mutations
+19
UPD 1q
+2q
+1p,4p,6,7,11,12,17
UPD 8p,4q,
16,18q
+20q
+2, +3, +4p, +4, +5, +5q, +8, +9,
+10, +11, +14q, +17
−1p, −1q, −4q, −6q, −8p, −13q, −15q, −16p, −16q, −18p, −18q,
−20q, −21q, −Xp, −Xq
CHC1704T, Male, 43yo, Africa
HCC, F2–F3, 14cm, Hepatitis.B
Drivers: TERT, TP53, EPHA4
Mutational signatures
24
5
16
HBV insertion TERT
TP53 R249S, EPHA4 F154L
31377 clonal mutations
2560 subclonal mutations
+5p,7q,6p,
8,11,14q
−1p, −3p, −4p, −4q, −6q, −10p, −10q, −11q, −13q, −14q, −16p,
−16q, −17p, −22q
CHC1717T, Male, 50yo, Africa
HCC, F4, 55cm, Hepatitis.B
Drivers: TERT, RPL22
Mutational signatures
16
5
22
24
4
12
HBV insertion TERT
RPL22 K15fs
17130 clonal mutations
903 subclonal mutations
+1q,5,7,10,
12,14q,UPD X
−4q
CHC1754T, Male, 34yo, Africa
HCC, F2–F3, 17cm, Hepatitis.B
Drivers: TERT, TP53, AXIN1, ALB
Mutational signatures
24
4
5
TP53 R249S, TERTp mut
ALB del, AXIN1 del
20190 clonal mutations
1268 subclonal mutations
Whole genome
duplication
+15q
−2p, −2q, −3p, −3q, −4q, −5q, −8p, −9p, −9q, −13q, −14q, −16p,
−16q, −17p, −19p, −21q
CHC2103T, Male, 56yo, Europe
HCC, F0–F1, 3.8cm, Alcohol.Intake – Hepatitis.B – Hepatitis.C – Tobacco
Drivers: CTNNB1, ACVR2A, RPS6KA3
Mutational signatures
5
4
16
12
17
CTNNB1 T41A, RPS6KA3 Y97*
ACVR2A del
14358 clonal mutations
3140 subclonal mutations
+1q,8q
+8p
−8p, −10q, −14q, −15q, −21q

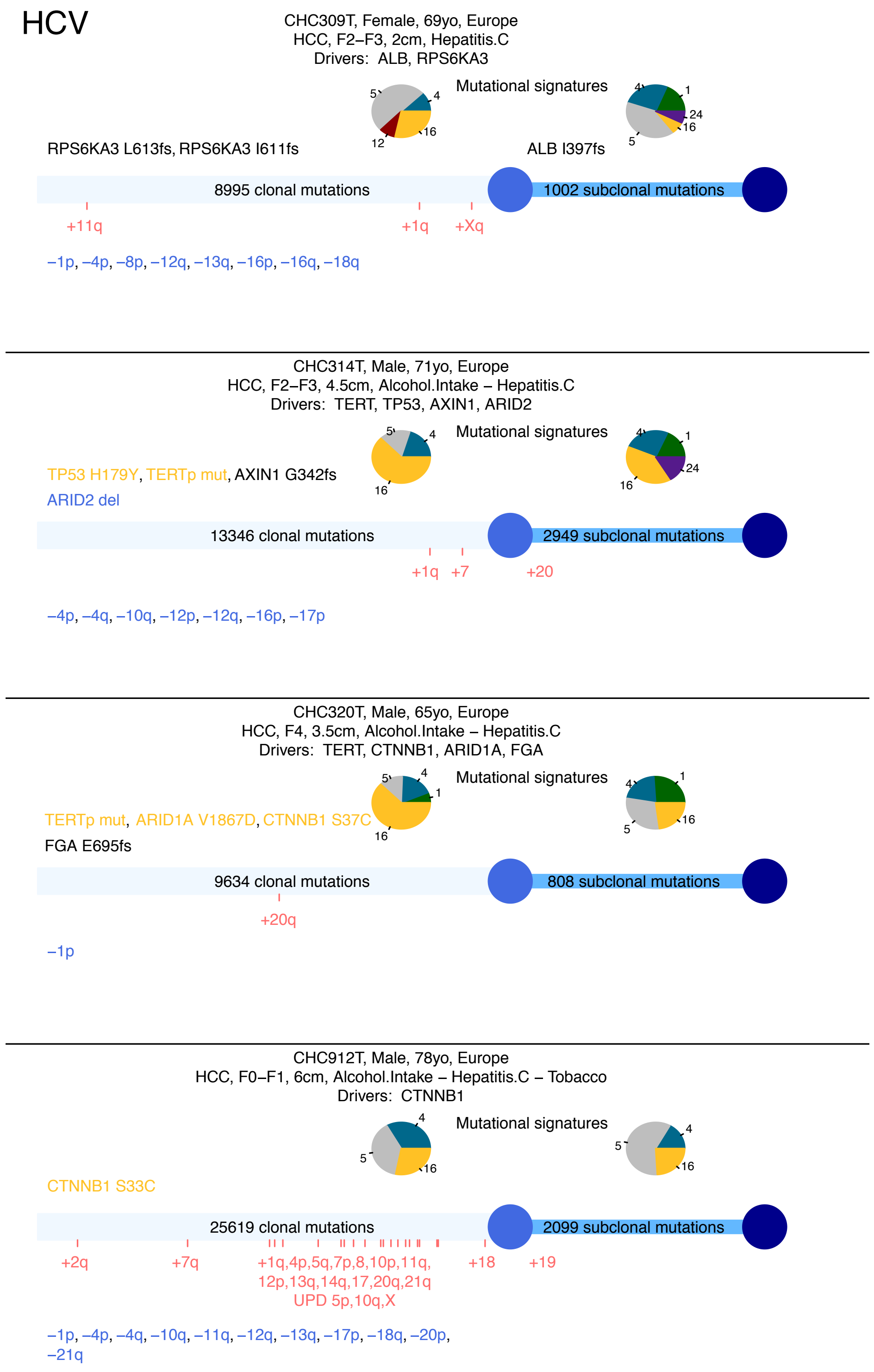
HCV
CHC309T, Female, 69yo, Europe
HCC, F2–F3, 2cm, Hepatitis.C
Drivers: ALB, RPS6KA3
Mutational signatures
RPS6KA3 L613fs, RPS6KA3 I611fs
ALB I397fs
8995 clonal mutations
1002 subclonal mutations
+11q
+1q
+Xq
−1p, −4p, −8p, −12q, −13q, −16p, −16q, −18q
CHC314T, Male, 71yo, Europe
HCC, F2–F3, 4.5cm, Alcohol.Intake – Hepatitis.C
Drivers: TERT, TP53, AXIN1, ARID2
Mutational signatures
TP53 H179Y, TERTp mut, AXIN1 G342fs
ARID2 del
13346 clonal mutations
2949 subclonal mutations
+1q
+7
+20
−4p, −4q, −10q, −12p, −12q, −16p, −17p
CHC320T, Male, 65yo, Europe
HCC, F4, 3.5cm, Alcohol.Intake – Hepatitis.C
Drivers: TERT, CTNNB1, ARID1A, FGA
Mutational signatures
TERTp mut, ARID1A V1867D, CTNNB1 S37C
FGA E695fs
9634 clonal mutations
808 subclonal mutations
+20q
−1p
CHC912T, Male, 78yo, Europe
HCC, F0–F1, 6cm, Alcohol.Intake – Hepatitis.C – Tobacco
Drivers: CTNNB1
Mutational signatures
CTNNB1 S33C
25619 clonal mutations
2099 subclonal mutations
+2q
+7q
+1q,4p,5q,7p,8,10p,11q,
12p,13q,14q,17,20q,21q
UPD 5p,10q,X
+18
+19
−1p, −4p, −4q, −10q, −11q, −12q, −13q, −17p, −18q, −20p,
−21q

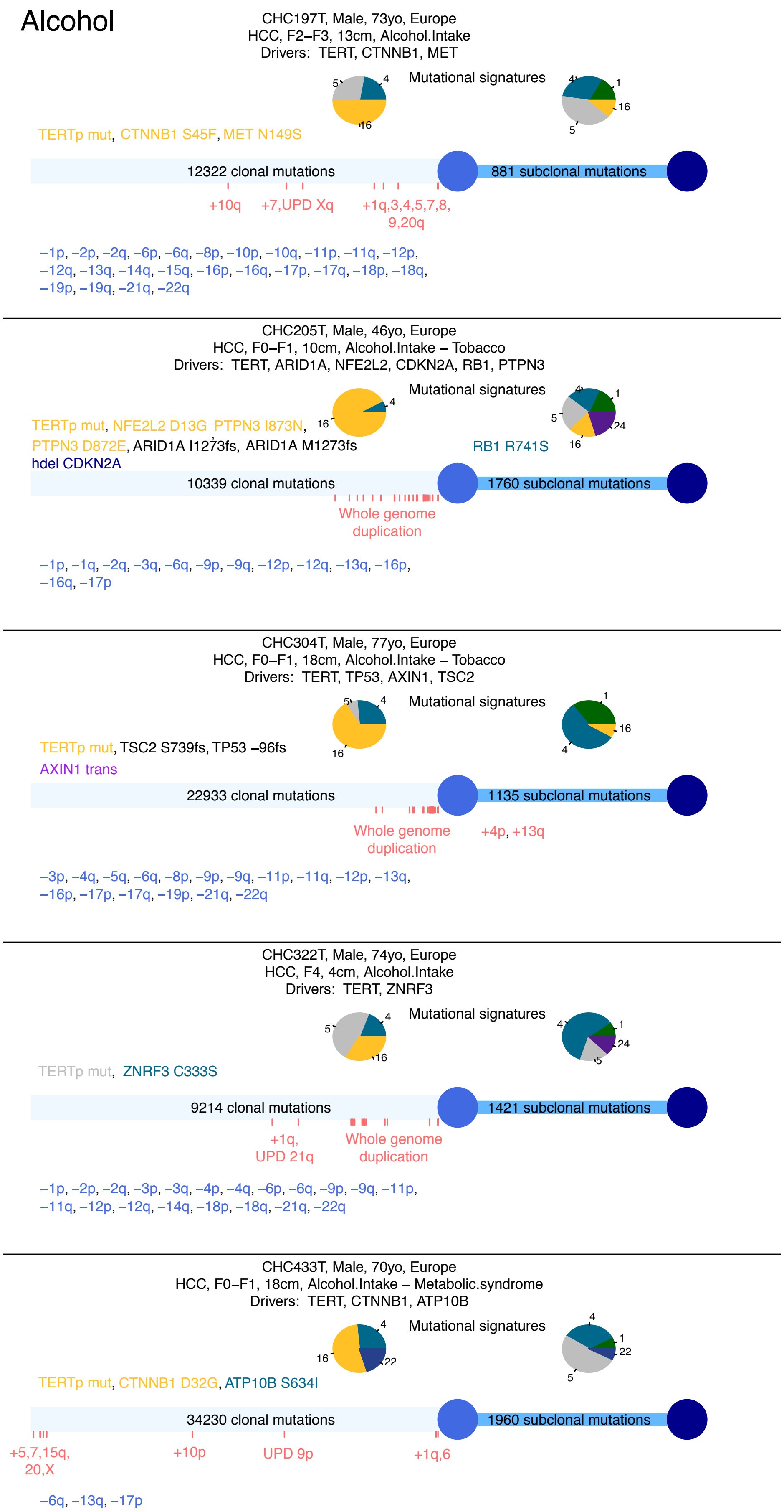
Alcohol
CHC197T, Male, 73yo, Europe
HCC, F2–F3, 13cm, Alcohol.Intake
Drivers: TERT, CTNNB1, MET
Mutational signatures
TERTp mut, CTNNB1 S45F, MET N149S
12322 clonal mutations
881 subclonal mutations
+10q
+7,UPD Xq
+1q,3,4,5,7,8,
9,20q
−1p, −2p, −2q, −6p, −6q, −8p, −10p, −10q, −11p, −11q, −12p,
−12q, −13q, −14q, −15q, −16p, −16q, −17p, −17q, −18p, −18q,
−19p, −19q, −21q, −22q
CHC205T, Male, 46yo, Europe
HCC, F0–F1, 10cm, Alcohol.Intake – Tobacco
Drivers: TERT, ARID1A, NFE2L2, CDKN2A, RB1, PTPN3
Mutational signatures
TERTp mut, NFE2L2 D13G PTPN3 I873N,
PTPN3 D872E, ARID1A I1273fs, ARID1A M1273fs
hdel CDKN2A
RB1 R741S
10339 clonal mutations
1760 subclonal mutations
Whole genome
duplication
−1p, −1q, −2q, −3q, −6q, −9p, −9q, −12p, −12q, −13q, −16p,
−16q, −17p
CHC304T, Male, 77yo, Europe
HCC, F0–F1, 18cm, Alcohol.Intake – Tobacco
Drivers: TERT, TP53, AXIN1, TSC2
Mutational signatures
TERTp mut, TSC2 S739fs, TP53 −96fs
AXIN1 trans
22933 clonal mutations
1135 subclonal mutations
Whole genome
duplication
+4p, +13q
−3p, −4q, −5q, −6q, −8p, −9p, −9q, −11p, −11q, −12p, −13q,
−16p, −17p, −17q, −19p, −21q, −22q
CHC322T, Male, 74yo, Europe
HCC, F4, 4cm, Alcohol.Intake
Drivers: TERT, ZNRF3
Mutational signatures
TERTp mut, ZNRF3 C333S
9214 clonal mutations
1421 subclonal mutations
+1q,
UPD 21q
Whole genome
duplication
−1p, −2p, −2q, −3p, −3q, −4p, −4q, −6p, −6q, −9p, −9q, −11p,
−11q, −12p, −12q, −14q, −18p, −18q, −21q, −22q
CHC433T, Male, 70yo, Europe
HCC, F0–F1, 18cm, Alcohol.Intake – Metabolic.syndrome
Drivers: TERT, CTNNB1, ATP10B
Mutational signatures
TERTp mut, CTNNB1 D32G, ATP10B S634I
34230 clonal mutations
1960 subclonal mutations
+5,7,15q,
20,X
+10p
UPD 9p
+1q,6
−6q, −13q, −17p

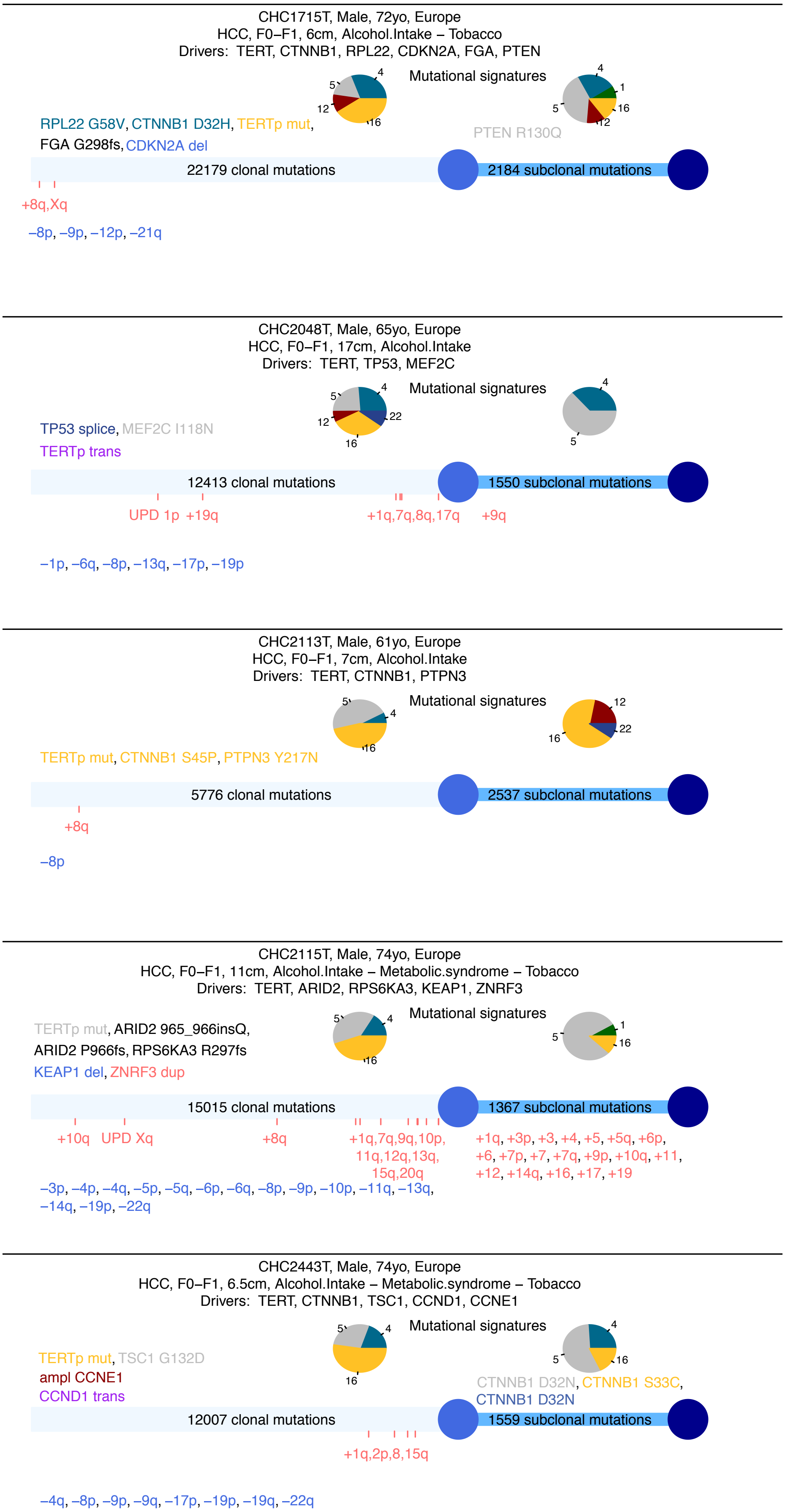
CHC1715T, Male, 72yo, Europe
HCC, F0–F1, 6cm, Alcohol.Intake – Tobacco
Drivers: TERT, CTNNB1, RPL22, CDKN2A, FGA, PTEN
Mutational signatures
RPL22 G58V, CTNNB1 D32H, TERTp mut,
FGA G298fs, CDKN2A del
PTEN R130Q
22179 clonal mutations
2184 subclonal mutations
+8q,Xq
–8p, –9p, –12p, –21q
CHC2048T, Male, 65yo, Europe
HCC, F0–F1, 17cm, Alcohol.Intake
Drivers: TERT, TP53, MEF2C
Mutational signatures
TP53 splice, MEF2C I118N
TERTp trans
12413 clonal mutations
1550 subclonal mutations
UPD 1p +19q
+1q,7q,8q,17q
+9q
–1p, –6q, –8p, –13q, –17p, –19p
CHC2113T, Male, 61yo, Europe
HCC, F0–F1, 7cm, Alcohol.Intake
Drivers: TERT, CTNNB1, PTPN3
Mutational signatures
TERTp mut, CTNNB1 S45P, PTPN3 Y217N
5776 clonal mutations
2537 subclonal mutations
+8q
–8p
CHC2115T, Male, 74yo, Europe
HCC, F0–F1, 11cm, Alcohol.Intake – Metabolic.syndrome – Tobacco
Drivers: TERT, ARID2, RPS6KA3, KEAP1, ZNRF3
Mutational signatures
TERTp mut, ARID2 965_966insQ,
ARID2 P966fs, RPS6KA3 R297fs
KEAP1 del, ZNRF3 dup
15015 clonal mutations
1367 subclonal mutations
+10q UPD Xq
+8q
+1q,7q,9q,10p,
11q,12q,13q,
15q,20q
+1q, +3p, +3, +4, +5, +5q, +6p,
+6, +7p, +7, +7q, +9p, +10q, +11,
+12, +14q, +16, +17, +19
–3p, –4p, –4q, –5p, –5q, –6p, –6q, –8p, –9p, –10p, –11q, –13q,
–14q, –19p, –22q
CHC2443T, Male, 74yo, Europe
HCC, F0–F1, 6.5cm, Alcohol.Intake – Metabolic.syndrome – Tobacco
Drivers: TERT, CTNNB1, TSC1, CCND1, CCNE1
Mutational signatures
TERTp mut, TSC1 G132D
ampl CCNE1
CCND1 trans
CTNNB1 D32N, CTNNB1 S33C,
CTNNB1 D32N
12007 clonal mutations
1559 subclonal mutations
+1q,2p,8,15q
–4q, –8p, –9p, –9q, –17p, –19p, –19q, –22q

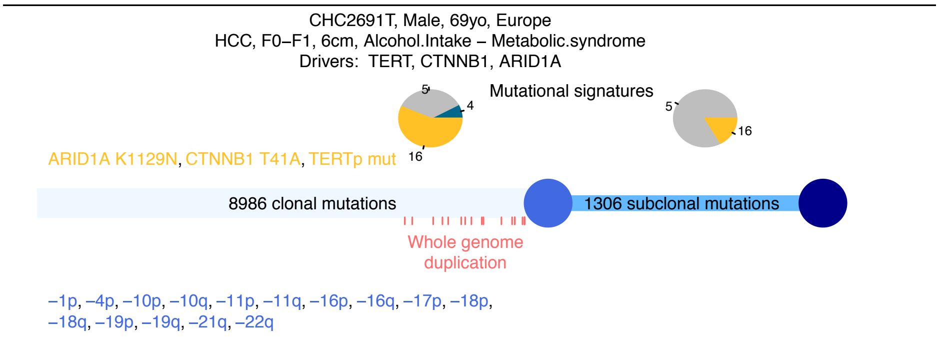
CHC2691T, Male, 69yo, Europe
HCC, F0–F1, 6cm, Alcohol.Intake – Metabolic.syndrome
Drivers: TERT, CTNNB1, ARID1A
Mutational signatures
5
4
16
5
16
ARID1A K1129N, CTNNB1 T41A, TERTp mut
8986 clonal mutations
1306 subclonal mutations
Whole genome duplication
−1p, −4p, −10p, −10q, −11p, −11q, −16p, −16q, −17p, −18p,
−18q, −19p, −19q, −21q, −22q

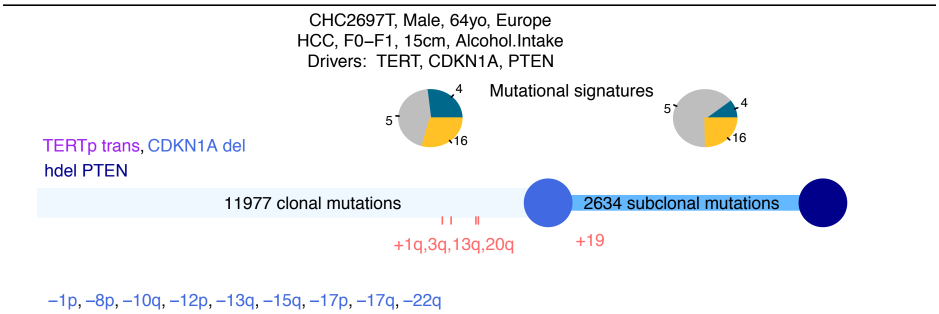
CHC2697T, Male, 64yo, Europe
HCC, F0–F1, 15cm, Alcohol.Intake
Drivers: TERT, CDKN1A, PTEN
Mutational signatures
4
5
16
5
4
16
TERTp trans, CDKN1A del
hdel PTEN
11977 clonal mutations
2634 subclonal mutations
+1q,3q,13q,20q
+19
−1p, −8p, −10q, −12p, −13q, −15q, −17p, −17q, −22q

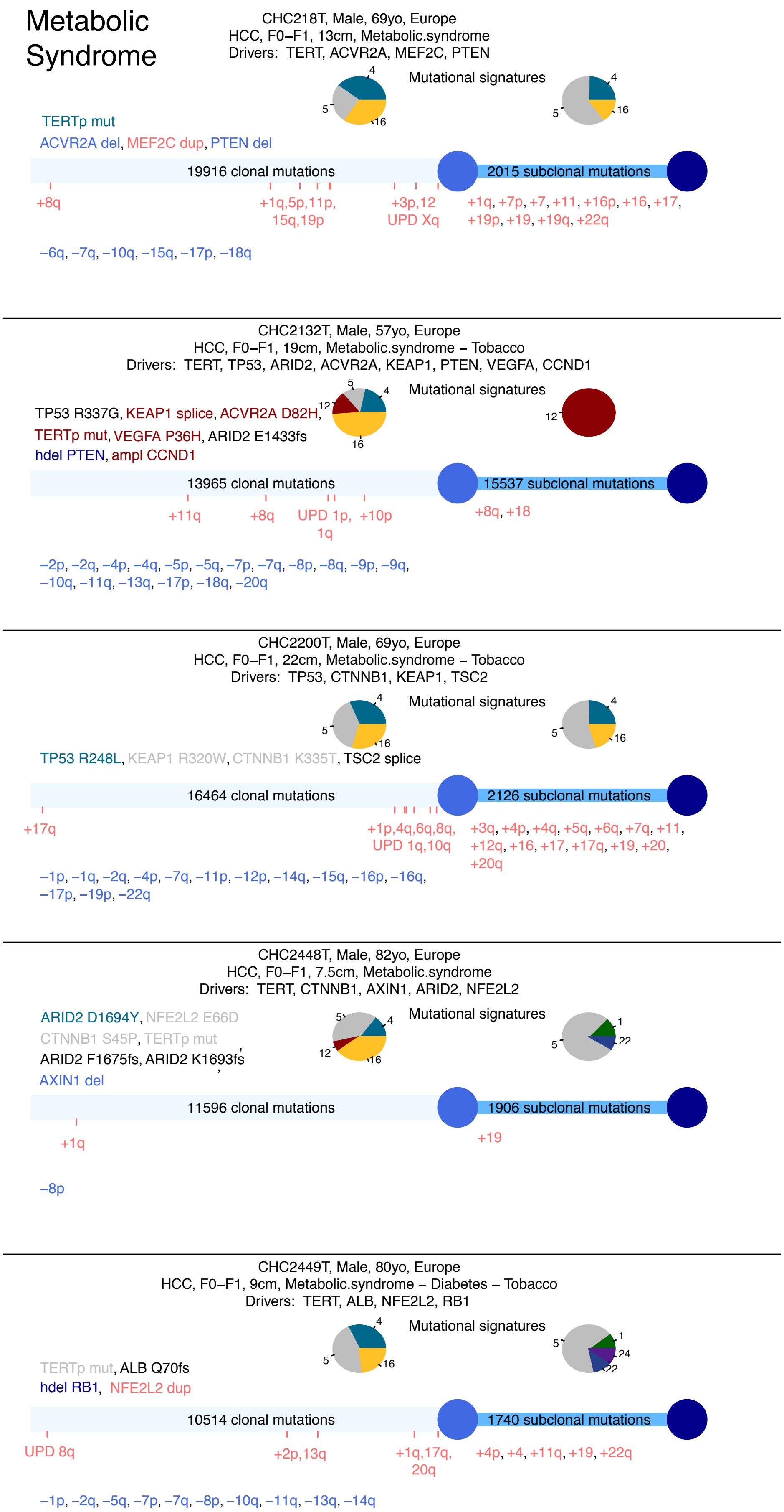
Metabolic Syndrome
CHC218T, Male, 69yo, Europe
HCC, F0−F1, 13cm, Metabolic.syndrome
Drivers: TERT, ACVR2A, MEF2C, PTEN
Mutational signatures
4
5
16
TERTp mut
ACVR2A del, MEF2C dup, PTEN del
19916 clonal mutations
2015 subclonal mutations
+8q
+1q,5p,11p, 15q,19p
+3p,12 UPD Xq
+1q, +7p, +7, +11, +16p, +16, +17, +19p, +19, +19q, +22q
−6q, −7q, −10q, −15q, −17p, −18q
CHC2132T, Male, 57yo, Europe
HCC, F0−F1, 19cm, Metabolic.syndrome − Tobacco
Drivers: TERT, TP53, ARID2, ACVR2A, KEAP1, PTEN, VEGFA, CCND1
Mutational signatures
5
4
12
16
TP53 R337G, KEAP1 splice, ACVR2A D82H,
TERTp mut, VEGFA P36H, ARID2 E1433fs
hdel PTEN, ampl CCND1
13965 clonal mutations
15537 subclonal mutations
+11q
+8q
UPD 1p, 1q
+10p
+8q, +18
−2p, −2q, −4p, −4q, −5p, −5q, −7p, −7q, −8p, −8q, −9p, −9q, −10q, −11q, −13q, −17p, −18q, −20q
CHC2200T, Male, 69yo, Europe
HCC, F0−F1, 22cm, Metabolic.syndrome − Tobacco
Drivers: TP53, CTNNB1, KEAP1, TSC2
Mutational signatures
4
5
16
TP53 R248L, KEAP1 R320W, CTNNB1 K335T, TSC2 splice
16464 clonal mutations
2126 subclonal mutations
+17q
+1p,4q,6q,8q, UPD 1q,10q
+3q, +4p, +4q, +5q, +6q, +7q, +11, +12q, +16, +17, +17q, +19, +20, +20q
−1p, −1q, −2q, −4p, −7q, −11p, −12p, −14q, −15q, −16p, −16q, −17p, −19p, −22q
CHC2448T, Male, 82yo, Europe
HCC, F0−F1, 7.5cm, Metabolic.syndrome
Drivers: TERT, CTNNB1, AXIN1, ARID2, NFE2L2
Mutational signatures
5
4
12
16
1
22
ARID2 D1694Y, NFE2L2 E66D
CTNNB1 S45P, TERTp mut
ARID2 F1675fs, ARID2 K1693fs
AXIN1 del
11596 clonal mutations
1906 subclonal mutations
+1q
+19
−8p
CHC2449T, Male, 80yo, Europe
HCC, F0−F1, 9cm, Metabolic.syndrome − Diabetes − Tobacco
Drivers: TERT, ALB, NFE2L2, RB1
Mutational signatures
4
5
16
1
24
22
TERTp mut, ALB Q70fs
hdel RB1, NFE2L2 dup
10514 clonal mutations
1740 subclonal mutations
UPD 8q
+2p,13q
+1q,17q, 20q
+4p, +4, +11q, +19, +22q
−1p, −2q, −5q, −7p, −7q, −8p, −10q, −11q, −13q, −14q

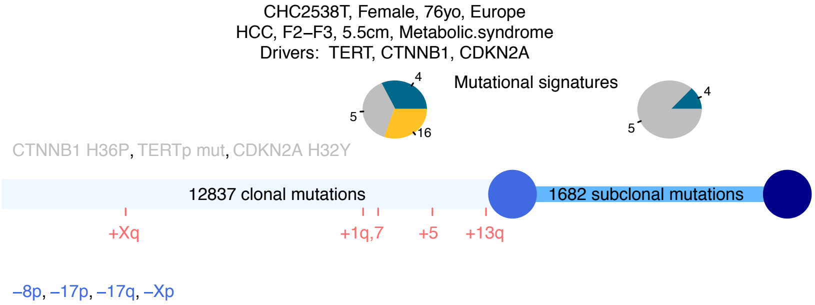

Hemochromatosis

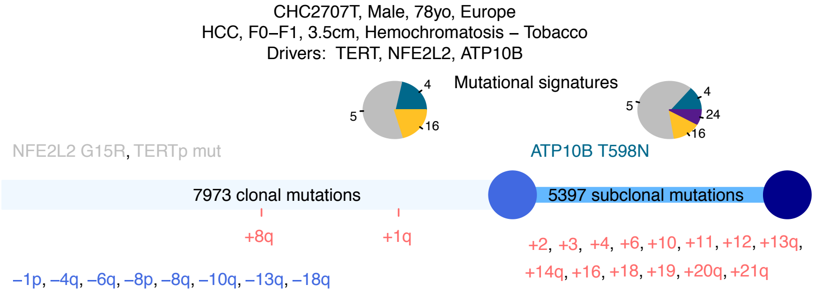

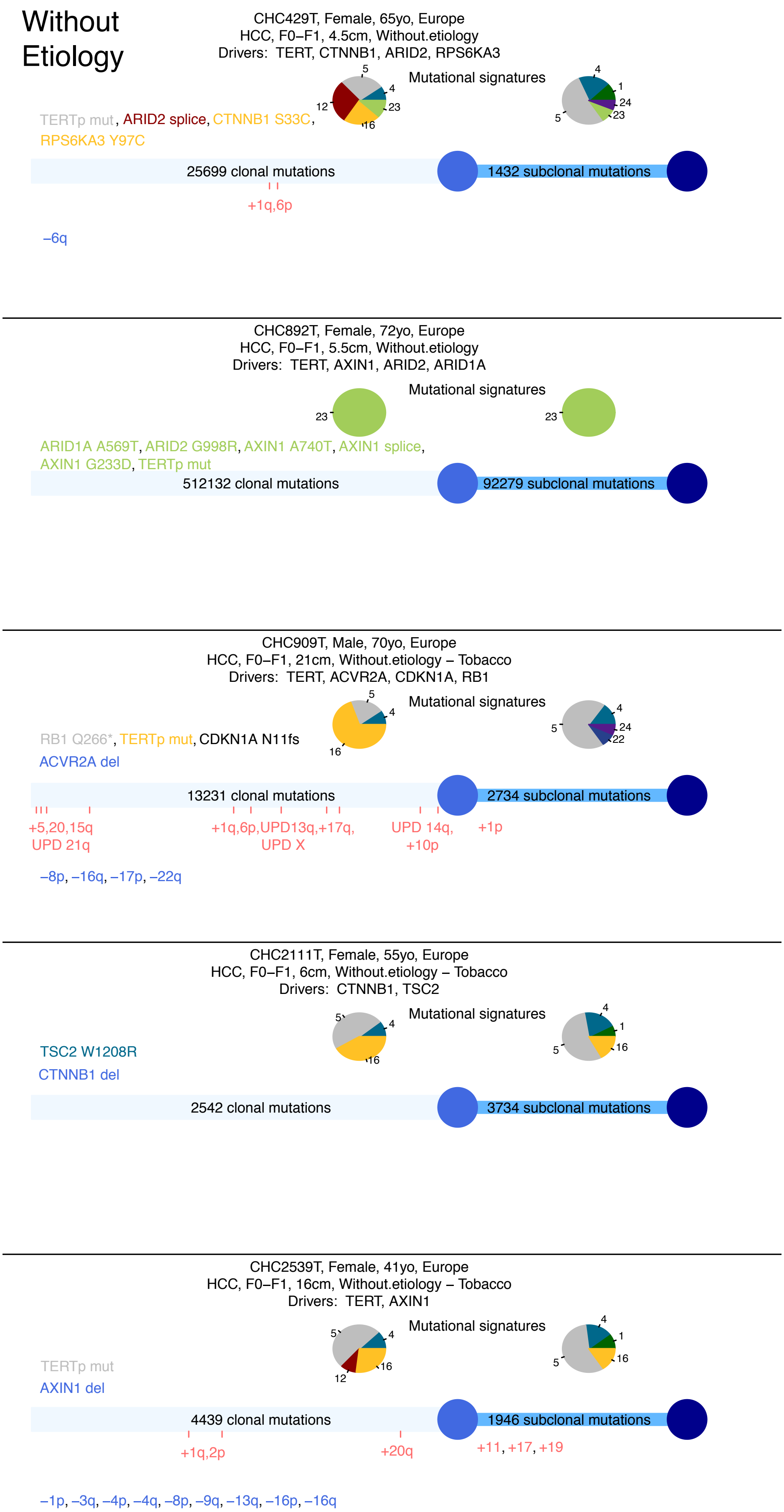
Without Etiology
CHC429T, Female, 65yo, Europe
HCC, F0−F1, 4.5cm, Without.etiology
Drivers: TERT, CTNNB1, ARID2, RPS6KA3
Mutational signatures
5
4
12
23
16
4
1
24
5
23
TERTp mut, ARID2 splice, CTNNB1 S33C,
RPS6KA3 Y97C
25699 clonal mutations
1432 subclonal mutations
+1q,6p
−6q
CHC892T, Female, 72yo, Europe
HCC, F0−F1, 5.5cm, Without.etiology
Drivers: TERT, AXIN1, ARID2, ARID1A
Mutational signatures
23
23
ARID1A A569T, ARID2 G998R, AXIN1 A740T, AXIN1 splice,
AXIN1 G233D, TERTp mut
512132 clonal mutations
92279 subclonal mutations
CHC909T, Male, 70yo, Europe
HCC, F0−F1, 21cm, Without.etiology − Tobacco
Drivers: TERT, ACVR2A, CDKN1A, RB1
Mutational signatures
5
4
16
4
5
24
22
RB1 Q266*, TERTp mut, CDKN1A N11fs
ACVR2A del
13231 clonal mutations
2734 subclonal mutations
+5,20,15q
UPD 21q
+1q,6p,UPD13q,+17q,
UPD X
UPD 14q,
+10p
+1p
−8p, −16q, −17p, −22q
CHC2111T, Female, 55yo, Europe
HCC, F0−F1, 6cm, Without.etiology − Tobacco
Drivers: CTNNB1, TSC2
Mutational signatures
5
4
16
4
1
5
16
TSC2 W1208R
CTNNB1 del
2542 clonal mutations
3734 subclonal mutations
CHC2539T, Female, 41yo, Europe
HCC, F0−F1, 16cm, Without.etiology − Tobacco
Drivers: TERT, AXIN1
Mutational signatures
5
4
16
12
4
1
5
16
TERTp mut
AXIN1 del
4439 clonal mutations
1946 subclonal mutations
+1q,2p
+20q
+11, +17, +19
−1p, −3q, −4p, −4q, −8p, −9q, −13q, −16p, −16q

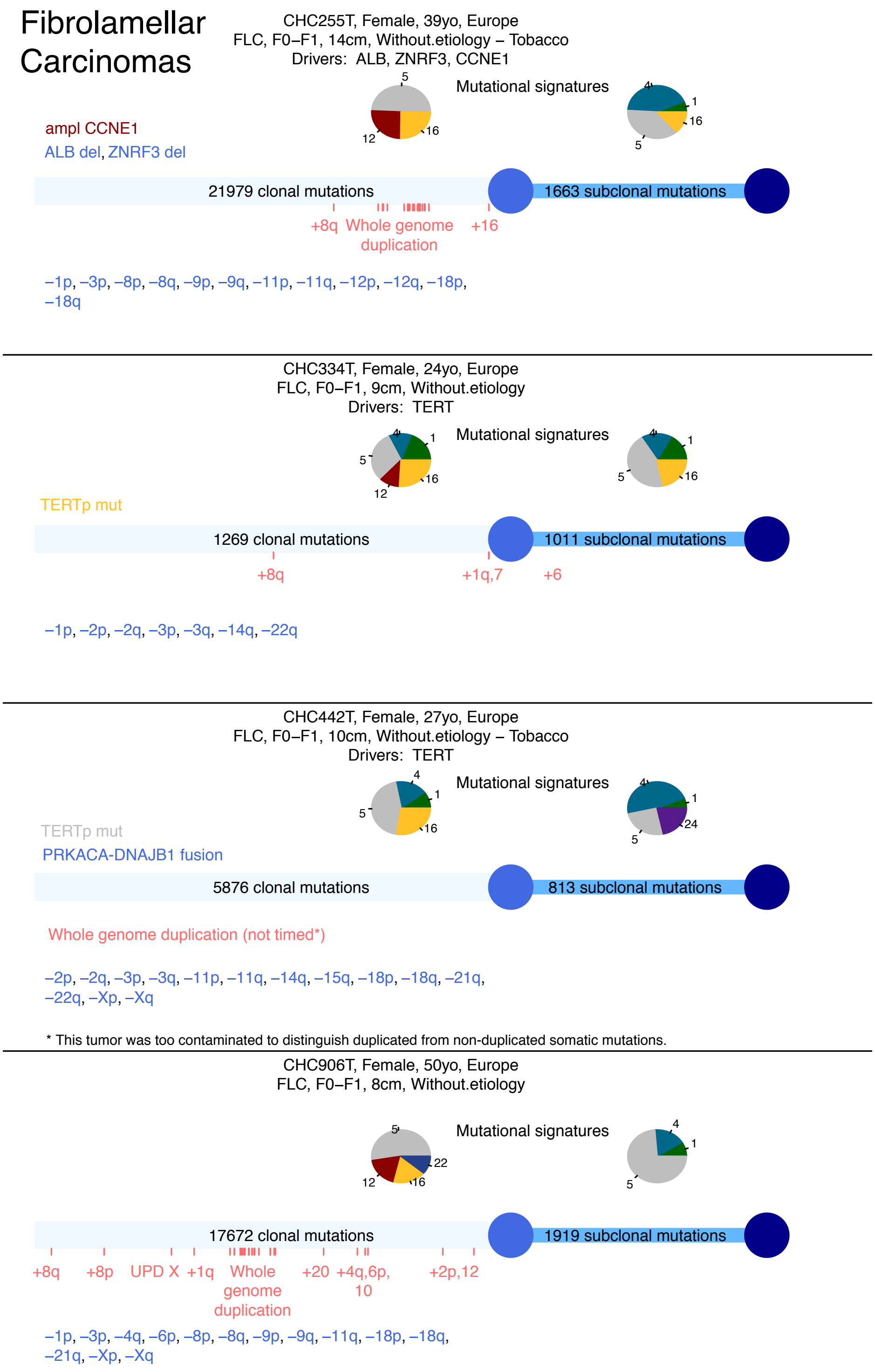
Fibrolamellar Carcinomas
CHC255T, Female, 39yo, Europe
FLC, F0–F1, 14cm, Without.etiology – Tobacco
Drivers: ALB, ZNRF3, CCNE1
Mutational signatures
5
12
16
4
1
16
5
ampl CCNE1
ALB del, ZNRF3 del
21979 clonal mutations
1663 subclonal mutations
+8q
Whole genome duplication
+16
–1p, –3p, –8p, –8q, –9p, –9q, –11p, –11q, –12p, –12q, –18p, –18q
CHC334T, Female, 24yo, Europe
FLC, F0–F1, 9cm, Without.etiology
Drivers: TERT
Mutational signatures
4
1
5
16
12
4
1
5
16
TERTp mut
1269 clonal mutations
1011 subclonal mutations
+8q
+1q,7
+6
–1p, –2p, –2q, –3p, –3q, –14q, –22q
CHC442T, Female, 27yo, Europe
FLC, F0–F1, 10cm, Without.etiology – Tobacco
Drivers: TERT
Mutational signatures
4
1
5
16
4
1
24
5
TERTp mut
PRKACA-DNAJB1 fusion
5876 clonal mutations
813 subclonal mutations
Whole genome duplication (not timed*)
–2p, –2q, –3p, –3q, –11p, –11q, –14q, –15q, –18p, –18q, –21q, –22q, –Xp, –Xq
* This tumor was too contaminated to distinguish duplicated from non-duplicated somatic mutations.
CHC906T, Female, 50yo, Europe
FLC, F0–F1, 8cm, Without.etiology
Mutational signatures
5
22
12
16
4
1
5
17672 clonal mutations
1919 subclonal mutations
+8q
+8p
UPD X
+1q
Whole genome duplication
+20
+4q,6p, 10
+2p,12
–1p, –3p, –4q, –6p, –8p, –8q, –9p, –9q, –11q, –18p, –18q, –21q, –Xp, –Xq

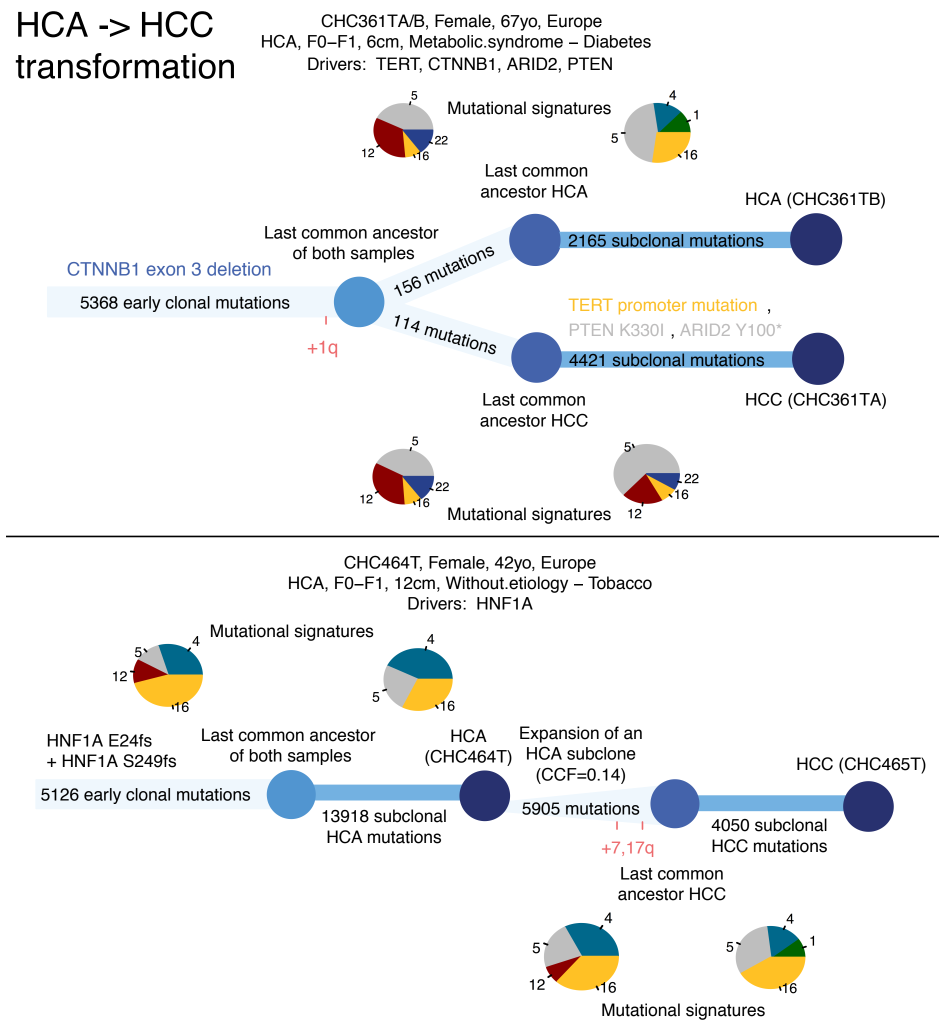
HCA -> HCC transformation
CHC361TA/B, Female, 67yo, Europe
HCA, F0–F1, 6cm, Metabolic.syndrome – Diabetes
Drivers: TERT, CTNNB1, ARID2, PTEN
Mutational signatures
Last common ancestor HCA
HCA (CHC361TB)
Last common ancestor of both samples
CTNNB1 exon 3 deletion
2165 subclonal mutations
156 mutations
5368 early clonal mutations
114 mutations
+1q
TERT promoter mutation ,
PTEN K330I , ARID2 Y100*
4421 subclonal mutations
Last common ancestor HCC
HCC (CHC361TA)
Mutational signatures
CHC464T, Female, 42yo, Europe
HCA, F0–F1, 12cm, Without.etiology – Tobacco
Drivers: HNF1A
Mutational signatures
HNF1A E24fs
+ HNF1A S249fs
Last common ancestor of both samples
HCA (CHC464T)
Expansion of an HCA subclone (CCF=0.14)
HCC (CHC465T)
5126 early clonal mutations
13918 subclonal HCA mutations
5905 mutations
+7,17q
4050 subclonal HCC mutations
Last common ancestor HCC
Mutational signatures

HCA

CHC605T, Female, 47yo, Europe
HCA, F0–F1, 6cm, Metabolic.syndrome – Diabetes
Drivers:

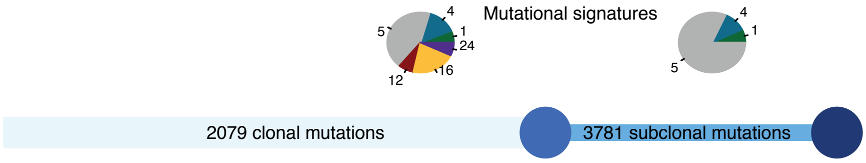

CHC2432T, Female, 43yo, Europe
HCA, F0–F1, 1.5cm, Without.etiology
Drivers:

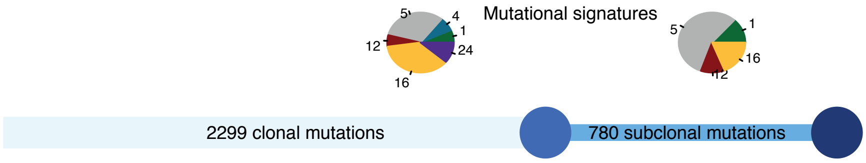

CHC2446T, Female, 45yo, Europe
HCA, F0–F1, 5cm, Without.etiology – Tobacco
Drivers:

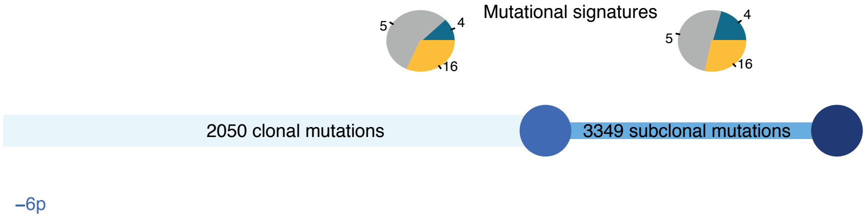

Legend

Tumor progression tree

- Last common ancestor
- Final tumor
- Clonal alterations
- Subclonal alterations

Mutational signatures

1 4 5 6
12 16 17
22 23 24

Copy-number and structural variations (SVs):

Deletions, Gains, Translocations
